# Visualizing Changes in *Cdkn1c* Expression Links Early-Life Adversity to Imprint Mis-regulation in Adults

**DOI:** 10.1016/j.celrep.2017.01.010

**Published:** 2017-01-31

**Authors:** Mathew Van de Pette, Allifia Abbas, Amelie Feytout, Gráinne McNamara, Ludovica Bruno, Wilson K. To, Andrew Dimond, Alessandro Sardini, Zoe Webster, James McGinty, Eleanor J. Paul, Mark A. Ungless, Paul M.W. French, Dominic J. Withers, Anthony Uren, Anne C. Ferguson-Smith, Matthias Merkenschlager, Rosalind M. John, Amanda G. Fisher

**Affiliations:** 1 MRC London Institute of Medical Sciences, Imperial College London, Hammersmith Hospital Campus, Du Cane Road, London W12 0NN, UK; 2 Photonics Group, Department of Physics, Blackett Laboratory, Imperial College London, London SW7 2AZ, UK; 3 Department of Genetics, University of Cambridge, Downing Street, Cambridge CB2 3EH, UK; 4 Cardiff School of Biosciences, Cardiff University, Cardiff CF10 3AX, UK

**Keywords:** imprinting, *Cdkn1c*, environmental stress, bioluminescence, luciferase reporter mice

## Abstract

Imprinted genes are regulated according to parental origin and can influence embryonic growth and metabolism and confer disease susceptibility. Here, we designed sensitive allele-specific reporters to non-invasively monitor imprinted *Cdkn1c* expression in mice and showed that expression was modulated by environmental factors encountered in utero. Acute exposure to chromatin-modifying drugs resulted in de-repression of paternally inherited (silent) *Cdkn1c* alleles in embryos that was temporary and resolved after birth. In contrast, deprivation of maternal dietary protein in utero provoked permanent de-repression of imprinted *Cdkn1c* expression that was sustained into adulthood and occurred through a folate-dependent mechanism of DNA methylation loss. Given the function of imprinted genes in regulating behavior and metabolic processes in adults, these results establish imprinting deregulation as a credible mechanism linking early-life adversity to later-life outcomes. Furthermore, *Cdkn1c-luciferase* mice offer non-invasive tools to identify factors that disrupt epigenetic processes and strategies to limit their long-term impact.

## Highlights

• Allele-specific expression of imprinted *Cdkn1c* imaged in vivo using bioluminescence
• Chromatin-modifying drugs applied in utero transiently de-repress *Cdkn1c* imprinting
• In utero exposure to low-protein diet permanently disrupts the *Cdkn1c* imprint
• Folate supplements during gestation protect against loss of *Cdkn1c* imprinting

## Graphical Abstract

**Figure:**
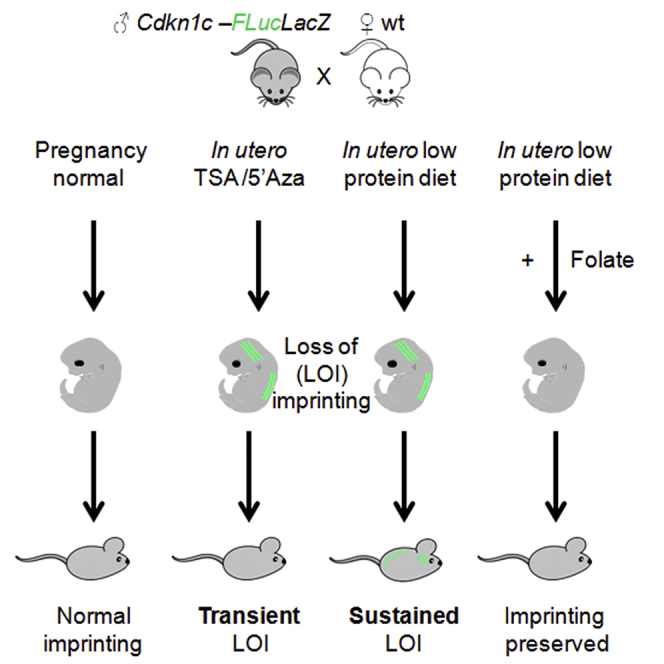

## Teaser

Van de Pette et al. use sensitive allele-specific reporters to longitudinally image imprinted *Cdkn1c* expression in mice and show that expression is modulated by environmental factors encountered in utero. These results establish imprinting deregulation as a mechanism linking early-life adversity to later-life outcomes and provide tools to detect imprinting changes in vivo.

## Introduction

Epigenetics is the study of heritable changes in gene expression that arise from non-genetic influences. Genomic imprinting is an epigenetic process found in eutherian and metatherian mammals that results in parent-of-origin-specific allelic expression (John and Surani, 2000). A relatively small subset of genes within the mammalian genome (0.4%) is imprinted (Surani et al., 1984, McGrath and Solter, 1984), and these show mono-allelic expression either universally or in specific tissues that favors the maternal (e.g., *Cdkn1c* and *Ube3a*) or the paternal allele (e.g., *Dlk1* and *Nnat*; Monk et al., 2006). Imprinted expression is initially determined by differential DNA methylation that is established in the germline (Surani, 1998). Although the rationale for genomic imprinting remains uncertain, the critical role of imprinted genes in embryonic growth, placental development, and neurogenesis (Cleaton et al., 2014) suggests that imprinting may serve to balance the selective pressures between parental genomes and control in utero offspring demand (Wolf and Hager, 2006, Day and Bonduriansky, 2004, Haig, 2004). Imprinted genes encode proteins that have a wide range of roles in nutrient transport, signaling, cell-cycle control, protein synthesis and degradation, and ion channel function. Their impact extends into postnatal life with key roles in the regulation of both metabolic and neuronal processes. Alterations at imprinted gene loci in humans are associated with rare disorders, such as Beckwith-Wiedemann syndrome (Lam et al., 1999), and also more common pathological conditions, including mental disability, impaired neuro-behavioral function, diabetes, obesity, muscle hypertrophy, and also with cancer (Radford et al., 2011).

Despite their importance, imprinted genes are particularly challenging to study. This in part reflects experimental difficulties that are common to mono-allelic genes, in that gene deletion experiments show all or nothing effects, whereas alterations in gene dosage can result in complex phenotypes in which isolating genetic and epigenetic traits is problematic (Cleaton et al., 2014, John, 2010). In addition, imprinted genes are often clustered within genomic domains in which regulation is achieved through multiple levels of epigenetic control, including DNA methylation, non-coding RNAs, and modified histones (Bartolomei and Ferguson-Smith, 2011). Finally, studies to assess the impact of chromatin-modifying drugs or environmental stress on imprinted gene expression require the maternal and paternal alleles to be discriminated on the basis of heterozygous SNPs or have used LacZ-based targeting of endogenous alleles (John, 2010). Whereas such approaches provide valuable generic tools to examine imprint dynamics through development and in response to stress, the invasive nature of the allelic readout means that it is not feasible to longitudinally monitor imprinted gene expression in different individuals throughout their life course. Models using fluorescence-based reporters have provided non-invasive readouts at whole-body and single-cell resolution (Jones et al., 2011, Swanzey and Stadtfeld, 2016), but tissue depth and sensitivity constraints may limit their general applicability. To provide new tools for investigating the consequences of early-life adversity, we generated a series of knockin embryonic stem cells (ESCs) and mouse lines in which *firefly luciferase* reports endogenous imprinted gene expression, and non-invasive bioluminescent imaging provides a means of monitoring expression longitudinally in vivo.

*Cdkn1c* is a maternally expressed imprinted gene that lies within the imprinting cluster 2 (IC2) on mouse chromosome 7 and is imprinted in both mice and humans (Hatada and Mukai, 1995, Hatada et al., 1996). The gene encodes a cyclin-dependent kinase inhibitor that is transiently expressed during embryogenesis in cells exiting proliferation (Lee et al., 1995, Matsuoka et al., 1995) and is particularly abundant within neural and skeleto-muscular tissue around mid-gestation (Westbury et al., 2001). *Cdkn1c* has an important role in regulating fetal growth and placental development (Andrews et al., 2007, Takahashi et al., 2000, Tunster et al., 2011) as well as lineage-specific roles, including in brown adipose tissue (Van De Pette et al., 2016), skeletal muscle (Osborn et al., 2011), and in adult quiescent stem cells (Zacharek et al., 2011, Matsumoto et al., 2011, Joseph et al., 2009). *Cdkn1c* lies within a complex imprinted domain regulated by an imprinting center that acquires DNA methylation in the maternal germline (gametic DMR; *KvDMR1*; John and Lefebvre, 2011, Hatada and Mukai, 1995, Hatada et al., 1996, Mohammad et al., 2012). This differentially methylated region spans the promoter of the paternally expressed long non-coding RNA *Kcnq1ot1* required for continuous domain-wide imprinting. The *Cdkn1c* promoter and gene body are also directly DNA methylated on the paternal allele post-fertilization, after allelic silencing has been established (somatic DMR; *Cdkn1c*-sDMR [somatic differentially methylated region]; Bhogal et al., 2004). Given the profound effect of modest dosage alteration of this gene on post-natal metabolic and behavioral processes (Andrews et al., 2007, Van De Pette et al., 2016, McNamara et al., 2016), it provides an ideal candidate to study using sensitive allele-specific reporters.

## Results

### Generating Luciferase-Based Allelic Reporters for Mouse *Cdkn1c*

**Figure 1:**
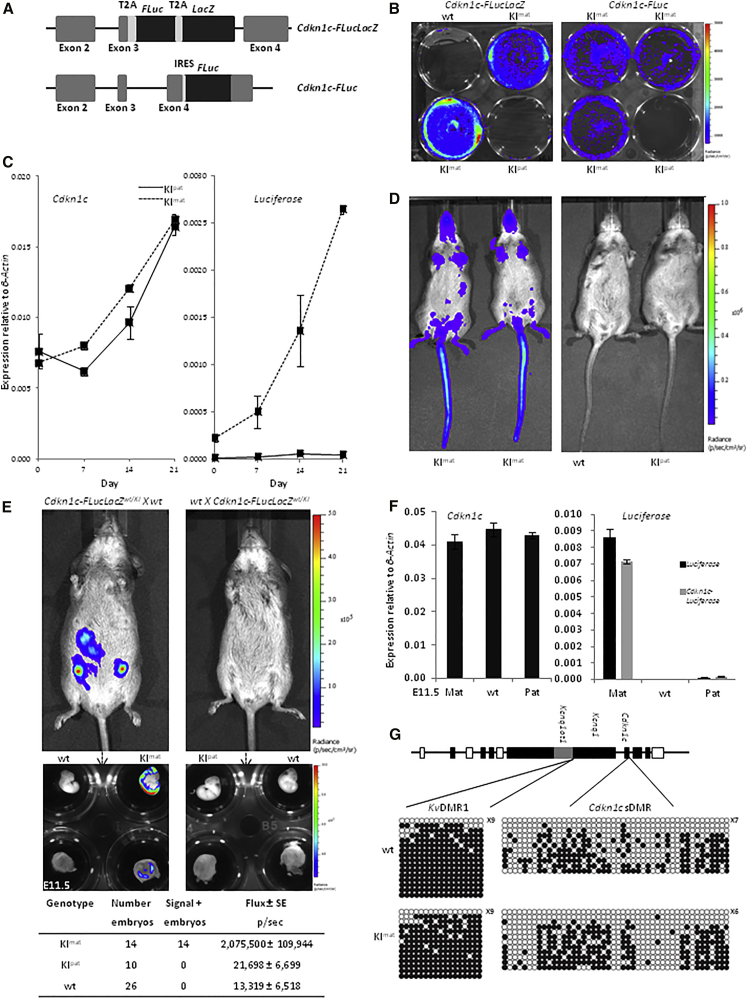
Visualizing *Cdkn1c* Gene Expression In Vivo Using Bioluminescence (A) Scheme of alternative knockin (KI) strategies used to generate *Cdkn1c-FLucLacZ* and *Cdkn1c-FLuc* embryonic stem cells (ESCs) and reporter mouse lines, in which sequences coding for the T2A peptide, the open reading frame of FLuc, a second T2A peptide, and the open reading frame of LacZ were inserted between the last amino acid and the translation termination codon in exon 3 (…KRLREGRG…; *Cdkn1c-FLucLacZ*) or IRES elements and the open reading frame of FLuc was inserted into a unique HindIII in the 3′ UTR (*Cdkn1c-FLuc*). (B) Low-level bioluminescence (blue-green) in *Cdkn1c-FLucLacZ* and *Cdkn1c-FLuc* ESCs was detected in clones with a presumed maternal insertion (KI^mat^), but not in clones with a paternal insertion (KI^pat^) or in wild-type ESCs (wt) (scale bar represents levels of bioluminescence). (C) Total *Cdkn1c* expression (left), determined by RT-PCR, was increased in ESC clones with either a KI^mat^ (dashed line) or a KI^pat^ (solid line) insertion over 21 days of embryoid body differentiation. *Luciferase* expression (right), determined by RT-PCR, was detected uniquely in KI^mat^ clones. Samples were normalized to β-actin and expressed as the mean ± SE. (D) Bioluminescent imaging of representative P28 female *Cdkn1c-FLucLacZ* mice. Luciferase activity was observed in *Cdkn1c-FLucLacZ* KI^mat^, with very low/negligible signals detectable upon paternal inheritance (KI^pat^) or in wild-type mice (wt). Strongest signal was evident in the skin, with low level signal detected in the internal organs. (E) Bioluminescence detected in pregnancies with maternal inheritance of *Cdkn1c-FLucLacZ* (KI^mat^, left) in utero, but not paternal inheritance (KI^pat^, right; less than twice background). Lower panels show bioluminescence imaging of dissected E11.5 embryos, where luciferase activity was seen in head and back of KI^mat^ embryos and placental tissue and quantified (flux). All *Cdkn1c-FLucLacZ* embryos imaged showed predicted parent-of-origin-specific bioluminescent activity. (F) Total *Cdkn1c* gene expression in embryos (E11.5) was determined by RT-PCR, and levels were similar in samples from wild-type and where *Cdkn1c-FLucLacZ* was transmitted maternally (KI^mat^) or paternally (KI^pat^; left). *Luciferase* (black) and *Cdkn1c-Luciferase* (gray) transcripts were detected uniquely from KI^mat^. Samples were normalized to β-actin and expressed as the mean ± SE. (G) Scheme of the mouse IC2 imprinting domain, showing the two DMRs that regulate *Cdkn1c* imprinted expression (*Kv*DMR1 and *Cdkn1c* sDMR) and the position of bi-allelic (white), maternally expressed (dark gray), and paternally expressed (light gray) genes. Bisulfite analysis showing DNA methylation at *Kv*DMR1 and *Cdkn1c* sDMR is similar in KI^mat^ and wt embryos at E11.5 (closed circles, methylated; open circles, un-methylated; where number indicates fully un-methylated strands).

Mouse ESC lines were generated in which *firefly luciferase* (*FLuc*) alone, or in combination with *β-galactosidase* (*FLucLacZ*), was knocked into the endogenous *Cdkn1c* locus (Figures S1A and 1A, respectively). In some of the resulting targeted clones, low-level bioluminescence was detected after adding the luciferase substrate D-luciferin, consistent with insertion of *luciferase* into the maternal allele in selected clones (Figure 1B, blue). Upon differentiation, we observed increased expression of *Cdkn1c* (Figures 1C, left, and S1B, left), as anticipated from previous studies (Wood et al., 2010). In clones with a presumed maternal insertion, increased *Cdkn1c* expression was coupled to a corresponding increase in *luciferase* expression (Figures 1C and S1B). In clones with a presumed paternal insertion (KI^pat^), increased levels of *Cdkn1c* expression were not accompanied by *luciferase* expression (Figures 1C and S1B), consistent with maintenance of the silent imprint.

Mice were generated from targeted ESCs to test whether bioluminescence was observed in offspring (Figures 1D and S1C) and to verify that this activity was transmitted in the appropriate parent-of-origin manner. Maternal transmission of the *FLucLacZ* transgene resulted in bioluminescent signal in the skin and internal organs of transgenic offspring (blue signal; KI^mat^) at 4 weeks of age, with no signal evident in offspring after paternal inheritance (KI^pat^) or in non-transgenic (wild-type [WT]) controls (Figure 1D). Strikingly, pregnant females carrying embryonic day 11.5 (E11.5) *Cdkn1c-FLucLacZ* KI^mat^ embryos (14/14), but not KI^pat^ embryos (0/10), showed a strong bioluminescent signal in the abdominal region (Figure 1E, upper). On dissection, transgenic embryos and placenta carrying the maternal targeted allele appropriately expressed luciferase, whereas those carrying the paternal targeted allele show no bioluminescence (Figure 1E, lower). Similar results were obtained with *Cdkn1c-FLuc* mice (Figure S1C).

Staining of E11.5 *Cdkn1c-FLucLacZ* KI^mat^ embryos for LacZ (Figure S1D) confirmed spatially appropriate expression in the hindbrain, spine, and developing cartilage, consistent with the published distribution of *Cdkn1c* (Westbury et al., 2001). This was further verified by 3D imaging using optical projection tomography (OPT) of cleared embryos (Figure S1D, lower; Movie S1), combined with photoacoustic tomography (Figure S1E). Importantly, no staining was detected in KI^pat^ embryos by this sensitive approach, confirming global repression of the paternal allele. Consistent with this, *luciferase* mRNA was only detectable after maternal inheritance (Figure 1F) alongside wild-type levels of the *Cdkn1c* transcript. Amplification with a primer set that spanned *Cdkn1c* exon 3 and *luciferase* exon 1 confirmed linked expression of *luciferase* and endogenous *Cdkn1c* transcripts (Figure 1F). Bisulfite analysis of the two differentially methylated regions associated with *Cdkn1c* imprinting (Bhogal et al., 2004, Mancini-Dinardo et al., 2006) showed normal DNA methylation patterns in heads of *Cdkn1c-FLucLacZ* KI^mat^ embryos (Figure 1G). Collectively, these data indicate that *luciferase* accurately reports *Cdkn1c* expression without impairing the methylation or regulation of the endogenous locus.

### Imprinted *Cdkn1c-FlucLacZ* Expression Is Appropriately Reset through the Germline

**Figure 2:**
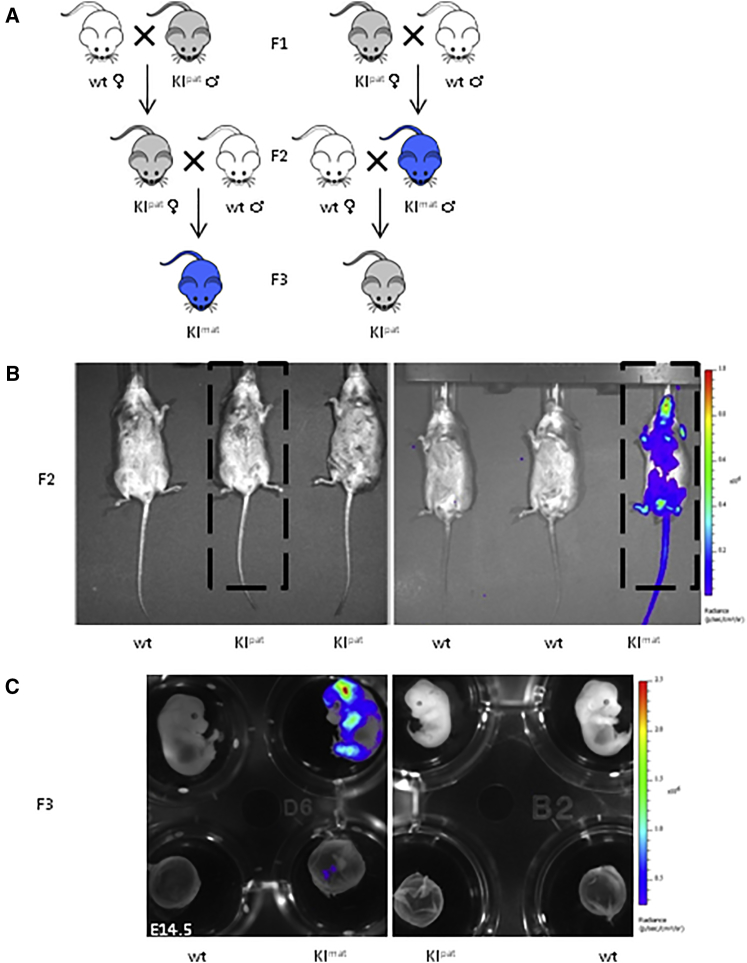
Correct Imprint Resetting of *Cdkn1c-FLucLacZ* across Generations (A) Diagram showing predicted expression and inheritance of a maternally expressed imprinted gene (such as *Cdkn1c*) or transgene (*Cdkn1c-FLucLacZ*) in reciprocal crosses across three generations. Wild-type mice are shown in white, expression through maternal inheritance is shown in blue, and inheritance of a silent imprint (*Cdkn1c-FLucLacZ*; KI^pat^) is indicated in gray. (B) Experimental evidence of imprint resetting; bioluminescent imaging of adult F2 mice, *Cdkn1c-FLucLacZ* KI^pat^ females (left box), and KI^mat^ males (right box), showing predicted parent-of-origin-specific luciferase activity (blue). Highlighted animals were then used to generate F3 (as outlined in A). (C) Bioluminescent image of E14.5 embryos, generated from the indicated transgenic parents; signal was detected upon maternal inheritance of *luciferase* in embryos (upper panel) and in placental tissue (lower panel; left), which had been silent in the previous generation. Conversely, paternal inheritance of the previously active luciferase was sufficient to silence the previously active allele (right).

Epigenetic marks that establish and maintain imprinting are normally erased and reset in the germline so that allelic expression is correctly maintained in subsequent generations (Bartolomei and Ferguson-Smith, 2011). To check whether erasure and resetting of imprints occurred normally in the *luciferase*-targeted mice, we tracked bioluminescence (blue) among reciprocal genetic crosses of *Cdkn1c-FLucLacZ* (gray) and wild-type mice (white) across generations (Figure 2A; F1, F2, and F3). Tracing bioluminescence activity across three generations revealed epigenetic inheritance as predicted (Figures 2B and 2C), in which allelic silencing of *Cdkn1c-FLucLacZ* was reversed through maternal transmission and re-established through paternal transmission. The ability to image *Cdkn1c* expression longitudinally in vivo through successive generations suggested that these reporter mice might be useful and robust models to screen for factors and environmental stresses that could interfere with imprinting. Importantly, as female mice inheriting *Cdkn1c-FLucLacZ* paternally (KI^pat^; left box, Figure 2B) were devoid of luciferase signal, these animals offered an optimal setting (minimal background) to detect bioluminescence signals in utero from KI^mat^ embryos and placental tissue (Figures 1E, left, and 2C).

### Chromatin-Modifying Drugs Transiently Disrupt Paternal Silencing of *Cdkn1c* In Utero

**Figure 3:**
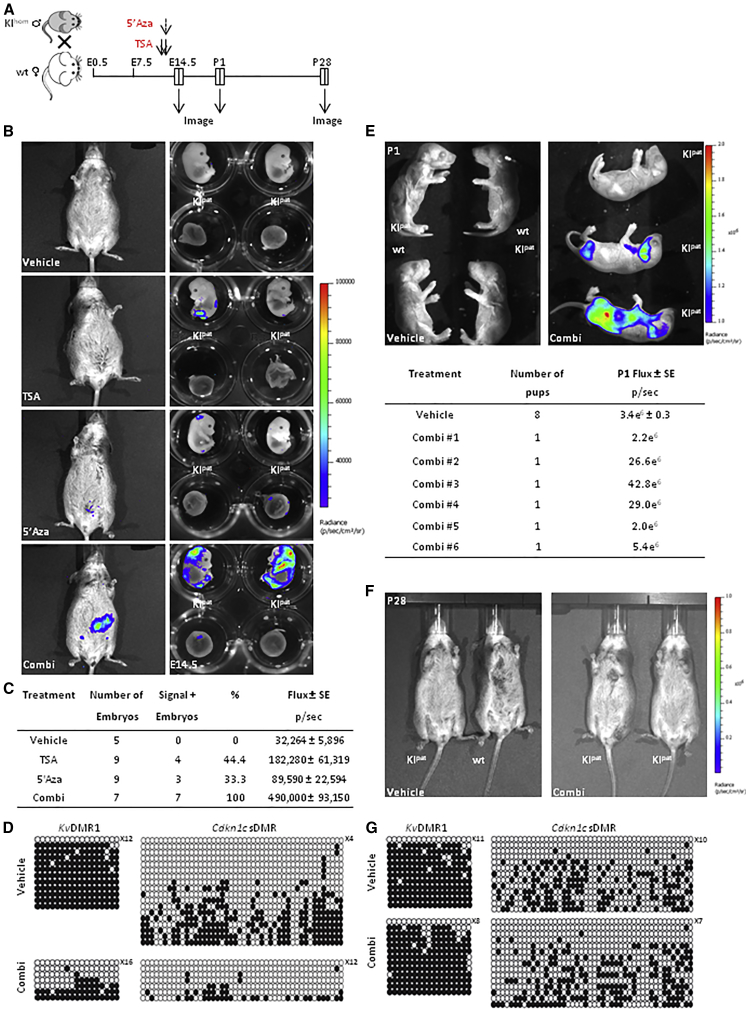
Silencing of Paternal *Cdkn1c-FLucLacZ* Is Transiently Released by In Utero Exposure to Epigenetic Drugs (A) Embryos carrying silent (paternally inherited) *Cdkn1c-FLucLacZ* were generated by mating wild-type (wt) females with homozygous *Cdkn1c-FLucLacZ* males. Pregnant females were treated with trichostatin A (TSA) or 5′ azacytidine (5′Aza) alone or together at the times indicated. Offspring were imaged at E14.5, at birth (P1), and at 4 weeks of age (P28). (B) Low-level bioluminescence was occasionally detected in 5′Aza- and TSA-alone treated pregnancies, whereas stronger and consistent signal (blue) was detected in combination-treated (Combi) embryos in utero (left) or individually dissected embryos (right) in the head and back. (C) *Cdkn1c-FLucLacZ*-derived bioluminescent activity was consistently elevated in E14.5 embryos exposed to combined drug treatment. (D) Bisulfite analysis of DNA methylation at the *Kv*DMR1 and *Cdkn1c* sDMR in the brain of E14.5 *Cdkn1c-FLucLacZ* KI^pat^ embryos shows reduced methylation in embryos exposed to combination drug treatment versus controls (closed circles, methylated; open circles, un-methylated). (E) Variable increases in luciferase activity (blue, flux) characterize combination-drug-treated *Cdkn1c-FLucLacZ* KI^pat^ animals at P1 (right), with no signal detected in vehicle-treated controls (left). (F) Luciferase activity was no longer detected in *Cdkn1c-FLucLacZ* KI^pat^ mice at P28 that had been exposed to combination drug treatment in utero. (G) Bisulfite analysis of DNA methylation at the *Kv*DMR1 and *Cdkn1c* sDMR in the brain of P28 *Cdkn1c-FLucLacZ* KI^pat^ mice shows that previously ablated methylation is restored by adulthood to normal levels (closed circles, methylated; open circles, un-methylated).

5′azacytidine (5′Aza) disrupts DNA methylation in cells by inhibiting DNMT1 activity, thereby preventing the incorporation of 5-methylcytosine into hemi-methylated DNA strands at S phase. In dividing cells in culture, 5′Aza treatment has been shown to reduce DNA methylation at the *Cdkn1c* promoter (Flotho et al., 2009). Trichostatin A (TSA) inhibits histone deacetylase activity and has been shown to deplete repressive histone marks at the *Cdkn1c* promoter (Yang et al., 2009). We reasoned that drugs that alter chromatin, such as 5′Aza and TSA, might be effective at disrupting *Cdkn1c* expression when epigenetic marks are consolidated (Bhogal et al., 2004, Umlauf et al., 2004). To examine this possibility, wild-type female mice were crossed with homozygous *Cdkn1c-FLucLacZ* (KI/KI) males to produce heterozygous offspring in which the *Cdkn1c-FLucLacZ* imprint was repressed. The pregnant females were then treated with drugs at E12.5–E13.5, and bioluminescence was evaluated at E14.5, at birth (P1), and at 4 weeks of age (postnatal day 28 [P28]; Figure 3A). Bioluminescence was detected in utero at E14.5 with strongest signal seen following combined drug treatment (Combi) (Figure 3B). Control vehicle-treated KI^pat^ embryos were consistently negative throughout these studies. For 5′Aza- (3/9) and TSA-treated (4/9) pregnancies, bioluminescence was not detected in all the transgenic embryos, whereas all the transgenic embryos (7/7) displayed increased luciferase activity upon combination treatment (Figure 3C). These animals showed a corresponding decrease in DNA methylation across the *Cdkn1c* somatic DMR at E14.5 as compared with controls (Figure 3D). We noticed that the levels of bioluminescence were generally lower than in age-matched *Cdkn1c-FLucLacZ* KI^mat^ embryos, consistent with partial de-repression of the paternal allele. Furthermore, de-repression appeared transient and was variable among combination drug-treated animals, as shown in pups imaged at birth (P1; Figure 3E). Four weeks after birth (P28), bioluminescence signal was no longer evident in drug-treated KI^pat^ animals (Figure 3F), and DNA methylation in the brain was similar in vehicle- and Combi-treated *Cdkn1c-FLucLacZ* KI^pat^ mice (Figure 3G). Taken together, these data show that conventional chromatin-modifying drugs alone or in combination are capable of relieving imprinted *Cdkn1c-FLucLacZ* repression in developing embryos.

### Dietary Protein Restriction In Utero Provokes De-repression of Paternal *Cdkn1c* into Adulthood

**Figure 4:**
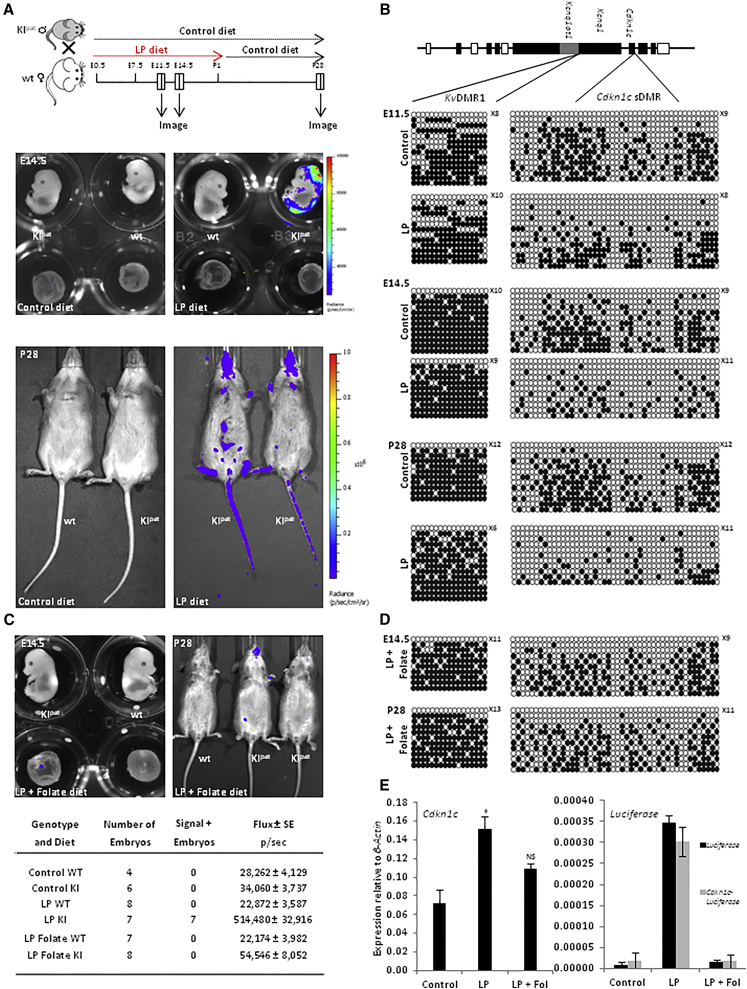
Stable Silencing of Paternally Inherited *Cdkn1c-FLucLacZ* during Life Course Depends upon the Availability of Methyl Donors In Utero (A) Offspring with a silent (paternally inherited) *Cdkn1c-FLucLacZ* were generated by mating wild-type (wt) females with heterozygous *Cdkn1c-FLucLacZ* males. Upon detection of a vaginal plug, a group of pregnant females were switched to a calorie-matched but low-protein (LP) diet for the duration of their pregnancy, with mothers and litters returning to a normal diet after birth. Pregnancies were imaged/examined at the times indicated (E11.5, E14.5, and P28). No mis-expression of luciferase was observed at day E11.5 (Figure S2), irrespective of diet; however, by E14.5, luciferase activity was detected selectively in embryos of mothers fed LP diet (upper right) and expression continued as these matured into adults (lower right). No signal was detected in animals fed normal (control) diet at any time. (B) Comparative bisulfite analysis of DNA methylation at the *Cdkn1c* locus in the brain of *Cdkn1c-FLucLacZ* KI^pat^ animals (E11.5, E14.5, and P28) born to mothers fed control versus LP diet during pregnancy. *Cdkn1c* sDMR becomes hypo-methylated in LP conditions in utero, and methylation is not restored subsequently (closed circles, methylated; open circles, un-methylated). Methylation at the *Kv*DMR1 is unaltered. (C) Pregnant females as in (A) were fed LP diet supplemented with increased folate. Bioluminescent imaging of embryos (E14.5) from mothers fed LP + folate showed reduced mis-expression of *Cdkn1c-FLucLacZ* KI^pat^ as compared with those fed LP alone (A), with luciferase activity remaining low or negligible as they matured into adults (P28; upper right; image scales same as A). (D) Bisulfite analysis showing DNA methylation at *Kv*DMR1 and *Cdkn1c* sDMR in E14.5 (upper) and P28 (lower) animals born to mothers fed LP + folate diet. Progressive hypo-methylation of the *Cdkn1c* sDMR was buffered against by the increased dietary folate. (E) Total *Cdkn1c* gene expression in E14.5 brain was determined by RT-PCR, and levels were elevated in samples from LP-exposed litters (p < 0.033), compared to control and LP + folate litters (p < 0.38). *Luciferase* (black) and *Cdkn1c-Luciferase* (gray) transcripts were detected using RT-PCR uniquely in LP brain samples, demonstrating loss of imprinting. Samples were normalized to β-actin and expressed as the mean ± SE.

*Cdkn1c* has previously been proposed to be sensitive to in utero dietary protein restriction (Vucetic et al., 2010). In particular, mice that were fed a low-protein diet through pregnancy (as a surrogate for early-life adversity) produced offspring with elevated levels of *Cdkn1c* in the midbrain associated with DNA hypo-methylation at the promoter. To examine whether exposure to low-protein diet in utero provokes de-repression of the silent paternal *Cdkn1c-FLucLacZ*, we crossed wild-type female mice with heterozygous *Cdkn1c-FLucLacZ* (WT/KI) males (Figure 4A, schematic). Pregnant mice were fed calorie-balanced, low-protein diet (LP) from the detection of vaginal plugs until birth. All newborn offspring were maintained thereafter on a normal (unrestricted) diet. This window of exposure ensures that the influence of LP diet is restricted to a specific period of development. Although bioluminescence signal was not detected at E11.5 (Figure S2), by E14.5, all *Cdkn1c-FLucLacZ* KI^pat^ embryos expressed luciferase following maternal exposure to low-protein diet (exemplified in Figure 4A, middle right). Signal was most pronounced in the head, and luciferase re-expression among KI^pat^ embryos was prominent in the midbrain region (Figure S3). De-repression was sustained in mice imaged subsequently at 4 weeks of age (Figure 4A, lower right) and throughout adulthood, despite no longer being exposed to a restricted diet. These data establish that in utero exposure to a low-protein diet results in permanent de-repression of the normally silent paternal allele.

To further explore the mechanism underlying *Cdkn1c* re-expression, we compared DNA methylation in the brain at E11.5, E14.5, and in adults at 4 weeks of age (Figure 4B). Appropriate DNA methylation at the somatic DMR was evident at E11.5, when no luciferase activity was detected (Figure S2), but this was progressively eroded in animals exposed to LP diet during gestation (Figure 4B). These data show that, under these conditions, the somatic DMR is established correctly, but not maintained, suggesting that dietary protein may be required to sustain DNA methylation at the paternal allele. In contrast, DNA methylation at *KvDMR1* was unaffected by LP diet (Figure 4B), consistent with previous reports (Ivanova et al., 2012).

### Rescue of Dietary-Induced Loss of Paternal *Cdkn1c* Silencing by Folate Supplementation

As dietary protein is known to be an important source of methyl donors required for DNA methylation, we hypothesized that a paucity of methyl donors might contribute to the failure to sustain repression of *Cdkn1c*^*pat*^ alleles in vivo. To test this, we repeated the dietary experiments using the low-protein diet with increased folate supplementation as a source of methyl donors. This had a dramatic effect, reducing paternal *Cdkn1c-FLucLacZ* bioluminescence to background levels in embryos (Figure 4C, left) and in resulting adults (Figure 4C, right). We also found that, following folate supplementation, methylation of *Cdkn1c* somatic DMR was indistinguishable from normal controls (Figures 4B and 4D). De-repression and restoration of *Cdkn1c-FlucLacZ* silencing in response to LP and LP + folate diet, respectively, was directly validated by allele-specific transcript analysis (Figure 4E). Thus, although previous studies have shown *Cdkn1c* upregulation in response to LP diet (Vucetic et al., 2010), our data now establish that dietary restriction can cause loss of imprinting.

## Discussion

In utero development is critically dependent on imprinted gene dose (Radford et al., 2011). This necessary control has been shown to extend into the programming of adult metabolism (Da Rocha et al., 2009; Charalambous et al., 2012). Here, we show that maternal dietary restriction has a profound impact on *Cdkn1c* expression in the embryo, provoking a partial loss of imprinting that persists through adult life, even when a normal diet is resumed. Prolonged exposure to low-protein diet during gestation erodes DNA methylation at the *Cdkn1c* somatic DMR and results in re-expression of the paternal allele. Because deregulation is rescued by elevated folate supplementation, methyl donor deprivation appears to be the most likely cause of imprint erosion. Although we do not yet know whether this reflects a specific window of vulnerability in embryonic development or simply an increased demand engendered by proliferating cells in the embryo, the observation that the gametic *Kv*DMR1 DMR resists DNA de-methylation supports previous findings that gametic differentially methylated regions (gDMRs) are relatively stable (Ivanova et al., 2012). Mechanistically, gametic and somatic DMRs both require the maintenance of DNA methylation by DNMT1 (Caspary et al., 1998, Bhogal et al., 2004). However, whereas zygotic deficiency of the de novo methylases *Dnmt3a* or *3b* has no effect on gDMR methylation, loss of *Dnmt3b* results in de-methylation of *Cdkn1c*-sDMR independent of *Kv*DMR1 status (Auclair et al., 2014). These data provide a precedent for the differential sensitivities of the gametic and somatic DMRs and implicate *Dnmt3b* as a candidate in preventing hypo-methylation at the *Cdkn1c sDMR*.

Imprinted genes are pivotal for regulating growth and metabolism, and yet the intricacies of imprinting have remained challenging to study. This reflects the intrinsic complexity of imprinting control regions (ICRs) but also a paucity of markers needed to reliably distinguish maternal from paternal alleles. Here, we describe two independent mouse lines in which luciferase-based bioluminescence reports allelic *Cdkn1c* expression, without disruption of endogenous gene output. The value of using this non-invasive approach is that it allows allelic expression to be imaged in individuals throughout life course so that epigenetic changes and their consequences can be evaluated directly. The close correspondence of luciferase expression in *Cdkn1c-FLuc* and *Cdkn1c-FLucLacZ* mouse lines suggests similar approaches might also be useful in studying allelic expression from other imprinted loci. Consistent with this idea, we have generated a series of ESC lines that report maternally expressed (*Ube3a*) or paternally expressed imprinted genes (*Dlk1*, *Nnat*, and *Igf2*) and are characterizing luciferase expression in mouse lines derived from such (Table S1). These lines, together with the *Cdkn1c-FLucLacZ* and *Cdkn1c-FLuc* lines described herein, provide novel genetic tools to interrogate the epigenetic mechanisms that establish, maintain, and reprogram imprinted gene expression in the female and the male germlines.

The observation that the *Cdkn1c* imprint is permanently disrupted by altered maternal diet provides a clear link between early-life adversity and the subsequent epigenetic mis-regulation in adult life. Our results suggest that a deficiency in methyl donor supply in utero is the most likely cause of imprint disruption, whereas limited exposure to well-characterized chromatin-modifying drugs in utero transiently deregulates imprint silencing. The basis of these different epigenetic outcomes is interesting and could reflect differences in the timing or length of exposure, inherent differences in cell proliferation, or the susceptibility of developing tissue to certain agents. Although future studies will be required to discriminate these possibilities, our ability to detect transient and permanent changes in imprint silencing in vivo offers an exciting new opportunity to explore the plasticity of epigenetic processes and their phenotypic outcome. More broadly, these luciferase-based imaging models will facilitate the rapid screening of epigenetic drugs and environmental stresses relevant for drug discovery programs and for understanding how epigenome deregulation in early life impacts upon longer-term health.

## Experimental Procedures

### Animal Maintenance

Mice were handled and all in vivo studies were performed in accordance with the United Kingdom Animals (Scientific Procedures) Act (1986), were approved by the Imperial College AWERB committee, and performed under a UK Home Office project license.

### Epidrug Injections

5′Aza (Sigma-Aldrich) and TSA (Sigma-Aldrich) were dissolved as 0.75 μg/μL and 0.3 μg/μL stocks in PBS and 30% ethanol, respectively. Wild-type 129S2/SvHsd dams were set up with *Cdkn1c-FLucLacZ* males, and upon vaginal plug discovery, matings were separated. For 5′Aza administration, pregnant dams were injected with 5 μg/g body weight at E12.5 intraperitoneally (i.p.). For TSA administration, pregnant dams were injected i.p. with 1 μg/g body weight at E12.5 and E13.5. Vehicle injections were performed with 30% ethanol at the same time points as TSA injections. Pregnant dams and embryos were imaged at E14.5; offspring were imaged at P1 and P28.

### Low Protein Study

Wild-type 129S2/SvHsd dams were set up with *Cdkn1c-FLucLacZ* males, and upon vaginal plug discovery, matings were separated. Females were fed either a low-protein chow (5769; TestDiet), a calorie-matched control chow (5755; TestDiet), or a low-protein chow with elevated folate supplement (5769 with 20 PPM Folate; TestDiet) until E11.5 or E14.5 for embryonic studies or birth for adult studies. Pregnant dams and embryos were imaged at E14.5; offspring were imaged at P28.

### Bioluminescent Imaging

D-Luciferin (PerkinElmer) was dissolved in H_2_0 at 30 mg/mL. For in vitro studies, cells were grown to 90% confluence, 150 μg/mL was added to the medium, and plates were imaged after 2 min. For in vivo studies, mice were weighed and injected i.p. with 0.15 mg/g body weight before being anesthetized with isoflurane. Mice were imaged 10 min post-injection in an IVIS Spectrum (PerkinElmer) under anesthesia. Images of cell plates, adult mice, and pregnant dams were taken at field of view (FOV) C, with binning 4 and 180 s exposure. For imaging of embryos, pregnant females were injected with D-Luciferin at least 10 min prior to imaging. Embryos were dissected into 24-well dishes containing PBS and placed in the IVIS Spectrum. Images of embryos were taken at FOV A, with binning 1, focus 1 cm, and 180 s exposure. For epidrug and low protein imaging, settings were the same, with the exception of binning 4 in embryos. No additional D-Luciferin was added, and imaging continued for up to 35 min post-injection. Analysis of images was performed on Living Image software (Caliper Life Sciences). For quantification of bioluminescent signal, regions of interest were drawn around embryos and signal flux within the region was calculated.

## Author Contributions

M.V.d.P., R.M.J., and A.G.F. conceived of and wrote the manuscript. R.M.J., A.C.F.-S., A.U., D.J.W., and M.M. were instrumental in designing the vectors used to generate and characterize the mice. A.S., J.M., and P.M.W.F. helped with the development of imaging protocols. A.F., L.B., W.K.T., A.D., G.M., A.A., E.J.P., M.A.U., and Z.W. contributed to the experiments described.

## Supplemental Information

Document S1. Supplemental Experimental Procedures, Figures S1–S3, and Table S1

Movie S1. OPT of LacZ-Stained E11.5 *Cdkn1c-FLucLacZ* Embryos, Related to Figures 1 and S1 Absorbance (green) was measured in the developing cartilage, spine, hindbrain and liver in KImat embryos. Weaker absorbance was also detectable in liver. Very low absorbance was observed in KIpat and wt embryos.

Document S2. Article plus Supplemental Information
