# Supplementary material for: Visualizing Changes in Cdkn1c Expression Links Early-Life Adversity to Imprint Mis-regulation in Adults
Source: Cell Rep. 2017 Jan 31;18(5):1090–9. doi: 10.1016/j.celrep.2017.01.010 (PMC5300902; doi:10.1016/j.celrep.2017.01.010)
Supplement: Document S1. Supplemental Experimental Procedures, Figures S1–S3, and Table S1 [file mmc1.pdf]

Cell Reports, Volume *18*

Supplemental Information

Visualizing Changes in *Cdkn1c* Expression Links Early-Life Adversity to Imprint Mis-regulation in Adults

Mathew Van de Pette, Allifia Abbas, Amelie Feytout, Gráinne McNamara, Ludovica Bruno, Wilson K. To, Andrew Dimond, Alessandro Sardini, Zoe Webster, James McGinty, Eleanor J. Paul, Mark A. Ungless, Paul M.W. French, Dominic J. Withers, Anthony Uren, Anne C. Ferguson-Smith, Matthias Merkenschlager, Rosalind M. John, and Amanda G. Fisher

Supplementary figures

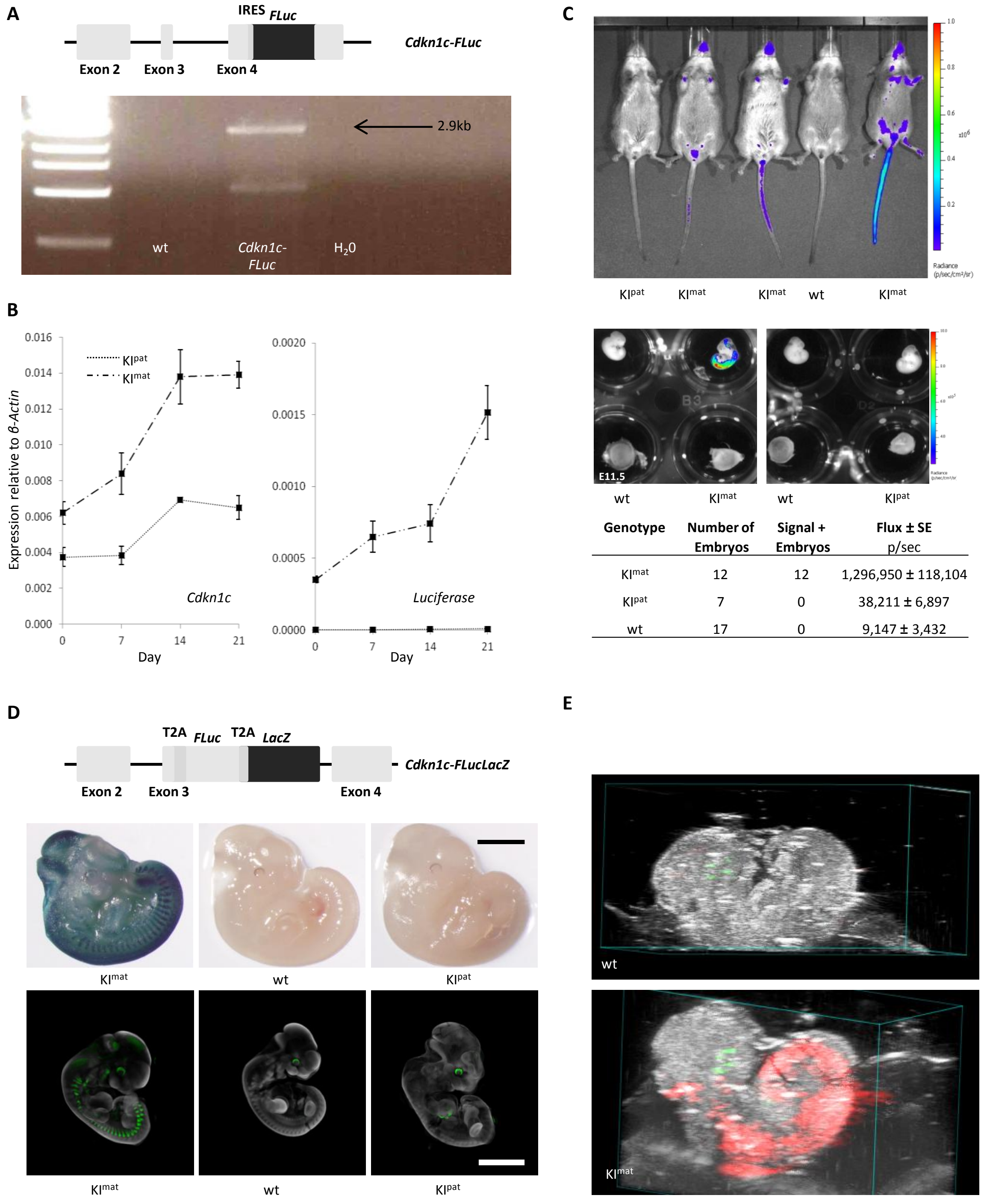

Figure S1. Characterization of *Cdkn1c-FLuc* and *Cdkn1c-FLucLacZ* lines, related to Figure 1.

(A) Construct designed to generate *Cdkn1c-FLuc* reporter ESCs, where insertion within the endogenous *Cdkn1c locus* was verified by long-range PCR and confirmed by DNA sequencing; 2.9 kb fragment corresponding to the *luciferase* transgene was detected in the targeted clone, not in wild type (wt) control cells. **(B)** Upon

differentiation to embryoid bodies total *Cdkn1c* transcript levels increased, as detected by RT-PCR, in clones that carried either a maternal (large dash) or a paternal (small dash) insertion. *Luciferase* transcripts, in contrast, were uniquely detected in ESC clones that had *Cdkn1c-FLuc* inserted into the maternal locus. **(C)** Bioluminescence imaging of representative P28 female *Cdkn1c-FLuc* mice shows signal (blue) in mice inheriting *luciferase* KI maternally (KI^{mat}), and no expression in wild type (wt) controls or animals with paternal inheritance (KI^{pat}). Bioluminescent signal (green, flux) in E11.5 embryos upon maternal inheritance but not paternal inheritance of *Cdkn1c-FLuc.* **(D)** Construct designed to generate *Cdkn1c-FLucLacZ* reporter ESCs (as in Figure 1A). LacZ staining of *Cdkn1c-FLucLacZ* embryos at E11.5 showing labeling of cartilage, spine and hind brain in KI^{mat} embryos, with no staining detected in KI^{pat} or wt embryos. Scale bar: 2 mm. Optical Projection Tomography (OPT) of LacZ stained E11.5 *Cdkn1c-FLucLacZ* embryos. Absorbance (green) was measured in the developing cartilage, spine, hindbrain and liver of KI^{mat} embryos. Very low absorbance was observed in KI^{pat} and wt embryos. Scale bar: 2 mm. Videos available of 3D reconstructions. **(E)** Photoacoustic imaging of LacZ stained E11.5 wt and KI^{mat} *Cdkn1c-FLucLacZ* embryos; signal (red) overlays ultrasound (grey) and shows detection in cartilage, spine, hind brain and liver.

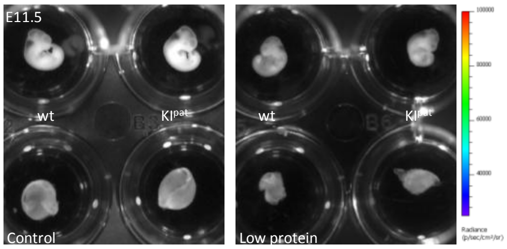

Genotype and Diet	Number of Embryos	Number+ Embryos	Flux ± SE p/sec
Control WT	6	0	14,395 ± 5,229
Control KI	5	0	26,438 ± 5947
Low protein WT	6	0	12,812 ± 7,680
Low protein KI	8	0	28,280 ± 7,908

Figure S2. Silencing of paternal *Cdkn1c-FLucLacZ* is retained at E11.5 in embryos exposed to LP diet *in utero*, related to Figure 4.

Cdkn1c-FLucLacZ KI^{pat} embryos were generated as in Figure 4A, with mothers receiving either normal (control) or low protein (LP) diet throughout pregnancy. Luciferase activity remained low in all *Cdkn1c-FLucLacZ* KI^{pat} embryos at E11.5, consistent with correct imprinting of the paternal allele at this stage.

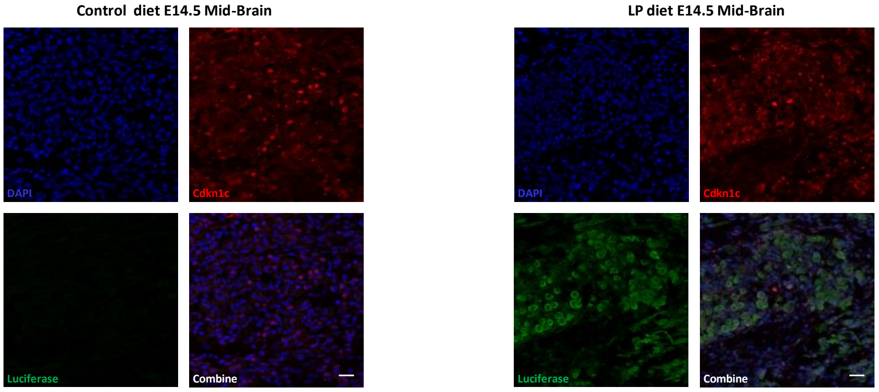

Figure S3. Low protein diet *in utero* disrupts *Cdkn1c* KI^{pat} silencing in regions of the brain, related to Figure 4.

(A) Cdkn1c (red) and luciferase (green) immunostaining in the E14.5 *Cdkn1c-FLucLacZ* KI^{pat} midbrain, where cell nuclei are highlighted by DAPI (blue). Embryos exposed to maternal low protein diet show loss of imprinting in the midbrain, with negligible signal detected in control embryos. Scale bar: 30 μm.

Table S1. Bioluminescent Reporters of Imprinted Gene Expression Models (BRIGEM) embryonic stem cell lines, related to Figure 1 and Figure S1.

Imprinted gene	Parental expression	ICR gametic methylation	Somatic DMR	ESC targeted line	No. of independent clones
Cdkn1c	Maternal	Maternal (*Kv*DMR1, *Kcnq1ot1* promoter)	*Cdkn1c* promoter (Paternal)	*Cdkn1c-T2A-FLuc-T2A-LacZ*	2
				Cdkn1c-IRES-Fluc	6
Dlk1	Paternal	Paternal (IG-DMR, intergenic)	No	*Dlk1-T2A-FLuc-T2A-LacZ*	1
Nnat	Paternal	Maternal (*Nnat* promoter)	No	*FLuc-T2A-LacZ-T2A-Nnat*	2
Igf2	Paternal	Paternal (*H19*-DMD, intergenic)	Igf2-DMR0 (Maternal, placenta only) Igf2-DMR1 (Paternal) Igf2-DMR2 (Paternal)	*Igf2-T2A-Luc2-T2A-LacZ*	2
Ube3a	Maternal (only in brain; bi-allelic in other tissues)	Maternal (*Snrpn*-CGI, *Snrpn* promoter)	No	*Ube3a-T2A-Luc2*	2

Video S1, related to Figure 1.

OPT of LacZ stained E11.5 *Cdkn1c-FLucLacZ* embryos. Absorbance (green) was measured in the developing cartilage, spine, hindbrain and liver in KI^{mat} embryos. Weaker absorbance was also detectable in liver. Very low absorbance was observed in KI^{pat} and wt embryos.

Supplemental Experimental Procedures

Generation of targeted ESCs and mice

The *Cdkn1c-IRES-luciferase* targeting sequence (*Cdkn1c-Fluc*) was generated in the pPGKneobpAlox2PGKDTA vector (Soriano's lab) by restriction fragment cloning of *IRES-Fluc* (Addgene) into the *Cdkn1c* 3'UTR (*HindIII* site) along with 2.9 kb of upstream sequence and 2.85 kb of *Cdkn1c* genomic sequence. Targeting was confirmed by long range PCR (Figure S1A). The *Cdkn1c-FLucLacZ* line was created by Taconic Biosciences and ESCs and animal founders were delivered to Imperial College. The ESC lines presented in Table S1 were also created by Taconic Biosciences.

ESC culture and embryoid body differentiation

ESCs were maintained in knockout DMEM (Gibco) supplemented with 10% foetal bovine serum (Gibco), penicillin/streptomycin, β-mercaptoethanol, L-Glutamine and LIF, on gelatinised plates, as previously described (Pereira et al., 2008). For embryoid body differentiation, cells were plated onto low adherence plates without LIF. Medium was changed every 1-2 days and samples were taken at the indicated time points.

Maintenance of mice

Mice were handled and all *in vivo* studies were performed in accordance with the United Kingdom Animals (Scientific Procedures) Act (1986), were approved by the Imperial College AWERB committee and performed under a UK Home Office project license. Mice were housed on a 12 hour light-dark cycle with a temperature range of 21 +/- 2°C in pathogen free conditions. *Cdkn1c-FLucLacZ* and *Cdkn1c-FLuc* lines were maintained on a 129S2/SvHsd background. For mating, males were set up with not more than three females and morning plug checking was performed. Upon plug discovery, females were considered E0.5.

Genotyping of animals

Genomic DNA was isolated from 4-week old ear biopsies or embryonic tails by digestion in lysis buffer (0.05 M Tris HCl pH 8, 0.025 M EDTA, 0.031% SDS, 0.02 M NaCl, 80 µg/ml Proteinase K (Sigma-Aldrich)) at 50°C with rocking. DNA was diluted 1:2 in 10 mM Tris HCl pH8 and 1 µl of diluted DNA was used in PCR analysis.

Beta-Galactosidase staining

E11.5 embryos were dissected and placed in cold LacZ fixative (2% formaldehyde, 0.2% glutaraldehyde, 0.02% Nonidet P-40, 1 mM $MgCl_2$, 0.1 mg/ml Sodium Deoxycholate in PBS) for 1 hour, kept at 4°C with rocking. Tissue was washed in PBS before being placed in LacZ stain (0.4 mg/ml X-Gal, 4 mM Potassium Ferrocyanide, 4 mM Potassium Ferricyanide, 1 mM $MgCl_2$, 0.02% Nonidet P-40 in PBS) for 4-6 hours at 4°C with rocking. Upon completion, embryos were washed twice in PBS before transfer to 70% ethanol and storage at 4°C. Photography was performed under standard light field conditions.

Optical projection tomography

LacZ stained E11.5 embryos (as above) were mounted in cylinders of 2% low melting point agarose. The mounted samples were dehydrated through graded methanol solutions and maintained in 100% methanol prior to clearing. They were subsequently immersed overnight in an optical clearing solution, BABB (1:2 Benzyl benzoate: Benzyl alcohol, Sigma Aldrich). Optical projection tomography (OPT) (Sharpe et al., 2002) was performed on a low-magnification imaging system. Briefly, the cleared samples were suspended from a rotation

stage (T-NM17A200, Zaber Technologies Inc) in a cuvette filled with BABB and imaged using a telecentric zoom lens (modules NT56-625, NT59-671 and NT59-672, Edmund Optics Ltd) with images recorded using a CCD camera operated at 2×2 pixel binning (Clara, Andor Technology Ltd). To measure the distribution of LacZ staining, transmitted light images were acquired every 1° over a full 360° sample rotation through a 716±20 nm band-pass filter (FF01-716/40-25, Laser 2000 UK Ltd). Average illumination and background images were also acquired. At each projection angle these images were combined to form an integrated absorption coefficient image, given by

$$\sum_i \alpha_i = \frac{1}{\Delta l} \ln\left|\frac{I_0 - I_b}{I - I_b}\right|$$

where $\sum_i \alpha_i$ is the sum of absorption coefficients from the voxels along a 'line-of-sight' (i.e. a projection), Δl is the linear size of a voxel (18.7 μm), I is the transmitted light image (i.e. image of the sample), I_0 is the average illumination image and I_b is the average background image. A filtered back-projection algorithm (Kak, 1988) was applied to this transformed dataset to produce a 3-D reconstruction of the absorption coefficient per voxel (displayed in green). In addition to the transmitted light data, fluorescence OPT acquisitions using a 473 nm excitation source (Cobolt Blues™, Cobolt AB) imaging at 520±17 nm (FF01-520/35-25, Laser 2000 UK Ltd) were also performed to reconstruct the whole sample volume (shown in greyscale).

Photoacoustic tomography

LacZ stained E11.5 embryos (as above, without clearing) were immersed in ultrasound gel and imaged by ultrasound and photoacoustic tomography with a Vevo-LAZR micro-ultrasound imaging system (FUJIFILM VisualSonics) using a 40 MHz centre frequency probe (LZ 550). The linear array transducer had a bandwidth of 32-55 MHz, axial resolution of 40 µm and lateral resolution of 90 µm. Photoacoustic spectra of the embryos were obtained by performing spectral scans from 680 nm to 970 nm with 5 nm step size. Three dimensional multi-spectral unmixing was performed using 680 nm, 720 nm, 750 nm, 800 nm and 850 nm laser wavelengths. For generation of stacks, a total volume of 8 mm was scanned, with z step size of 0.076 mm.

Immunofluorescence

Embryos were dissected and fixed for 4 hours in phosphate-buffered 4% paraformaldehyde. Samples were washed in PBS and transferred to 30% sucrose in PBS for cryopreservation. After cryopreservation, samples were embedded in OCT and stored at -80°C until use. Sections of 10 µm were cut with a cryostat (Leica) and applied to polysine slides (VWR). Circles were drawn around sections with a hydrophobic pen (Invitrogen) and sections were incubated in block solution (5% Donkey Serum, 0.1% Fraction V BSA, 0.1% Triton-X100 (all Sigma-Aldrich)) for 30 mins. After blocking, immunolabelling for Cdkn1c (1:200, KP39, SantaCruz) and Luciferase (1:250, L0159, Sigma-Aldrich) was carried out and detected using Alexa Fluor® conjugated secondary antibodies (Abcam). Slides were mounted with DAPI containing medium (Vector) and images were acquired using a Leica SP8 confocal microscope and LAS X software.

RNA extraction and RT-PCR analysis

RNA was extracted with RNA-Bee (Amsbio) and all RNA precipitation steps were performed with 100% ethanol. Reverse transcription was performed using Superscript III Reverse transcriptase (Invitrogen) as per the manufacturer's protocol, with minor modifications. RT-PCR was performed on a CFX96 Real-Time System (Bio-Rad) with QuantiTect SYBR Green Master Mix (Qiagen) as per the manufacturer's protocol. Samples were normalised to *β-Actin* and expressed as the mean ± standard error. Student's T-test was performed for statistical analysis of *Cdkn1*c expression in low protein study.

Bisulphite sequencing

Bisulphite modification of DNA was carried out with the EZ DNA Methylation Kit (ZymoGenetics) according to the manufacturer's recommendations. PCR primers that specifically recognize bisulphite-converted DNA were used to amplify regions spanning three imprinted DMRs. PCR products were separated on an agarose gel and bands corresponding to the predicted size were excised and cleaned up with a Gel Extraction kit (QIAquick, Qiagen). Ligation of product into pGEM-T Easy vector (Promega) was performed before transformation into DH5alpha cells. Cells were plated onto LB/Ampicillin/IPTG/X-Gal plates and grown up overnight at 37 °C.

Colonies were picked (24 per sample) and expanded in LB/Ampicillin broth overnight at 37°C. The following morning, plasmids were purified with the Wizard® SV 96 Plasmid DNA Purification System (Promega) according to the manufacturer's recommendations and sent for sequencing.

Bisulphite primers

Cdkn1c sDMR F: AGTATAATGTAGTATTTTTAGT
Cdkn1c sDMR R: AAAACTATACCCAACTCCATA

*Kv*DMR1 F: TAAGGTGAGTGGTTTAGGAT
*Kv*DMR1 OutR: AATCCCCCACACCTAAATTC
*Kv*DMR1 InR: CCACTATAAACCCACACATA

M13 R: CAGGAAACAGCTATGAC

Primers

5'Long Range *Cdkn1c-FLuc* F: CCAGGACCCAGCTGGTAGTA
5'Long Range *Cdkn1c-FLuc* R: AGGAACTGCTTCCTTCACGA

3'Long Range *Cdkn1c-Fluc* F: GCTTCTGAGGCGGAAAGAAC
3'Long Range *Cdkn1c-Fluc* R: GGGGCCTGAATTGCAACTTA

Cdkn1c-FLucLacZ KI GenoF: CTCCATGCGATCACAGTGG
Cdkn1c-FLucLacZ KI GenoR: CTTTGGATCCAGTGGACTGG

β-Actin F: CCTGTATGCCTCTGGTCGTA
β-Actin R: CCATCTCCTGCTCGAAGTCT

Luciferase F: GTTTTGGAGCACGGAAAGAC
Luciferase R: ACCTTTCGGTACTTCGTCCA

Cdkn1c F: AGAGAACTGCGCAGGAGAAC
Cdkn1c R: TCTGGCCGTTAGCCTCTAAA

Cdkn1c-FLuc F: AGAGAACTGCGCAGGAGAAC
Cdkn1c-FLuc R: GTTCCATCTTCCAGCGGATA

General experimental approaches

Where possible, investigators were blinded to the genotype of both study animals and that of tissue samples. Treatments were administered in random order, with all pharmacological and metabolic studies replicated in at least two independent cohorts.

KAK, A. C. S., M; 1988. Principles of computerized tomographic imaging. IEEE Press, New York.

PEREIRA, C. F., TERRANOVA, R., RYAN, N. K., SANTOS, J., MORRIS, K. J., CUI, W., MERKENSCHLAGER, M. & FISHER, A. G. 2008. Heterokaryon-Based Reprogramming of Human B Lymphocytes for Pluripotency Requires Oct4 but Not Sox2. *Plos Genetics,* 4.

SHARPE, J., AHLGREN, U., PERRY, P., HILL, B., ROSS, A., HECKSHER-SORENSEN, J., BALDOCK, R. & DAVIDSON, D. 2002. Optical projection tomography as a tool for 3D microscopy and gene expression studies. *Science,* 296**,** 541-545.
