# Supplementary figures and images for: Visualizing Changes in Cdkn1c Expression Links Early-Life Adversity to Imprint Mis-regulation in Adults
Source: Cell Rep. 2017 Jan 31;18(5):1090–9. doi: 10.1016/j.celrep.2017.01.010 (PMC5300902; doi:10.1016/j.celrep.2017.01.010)

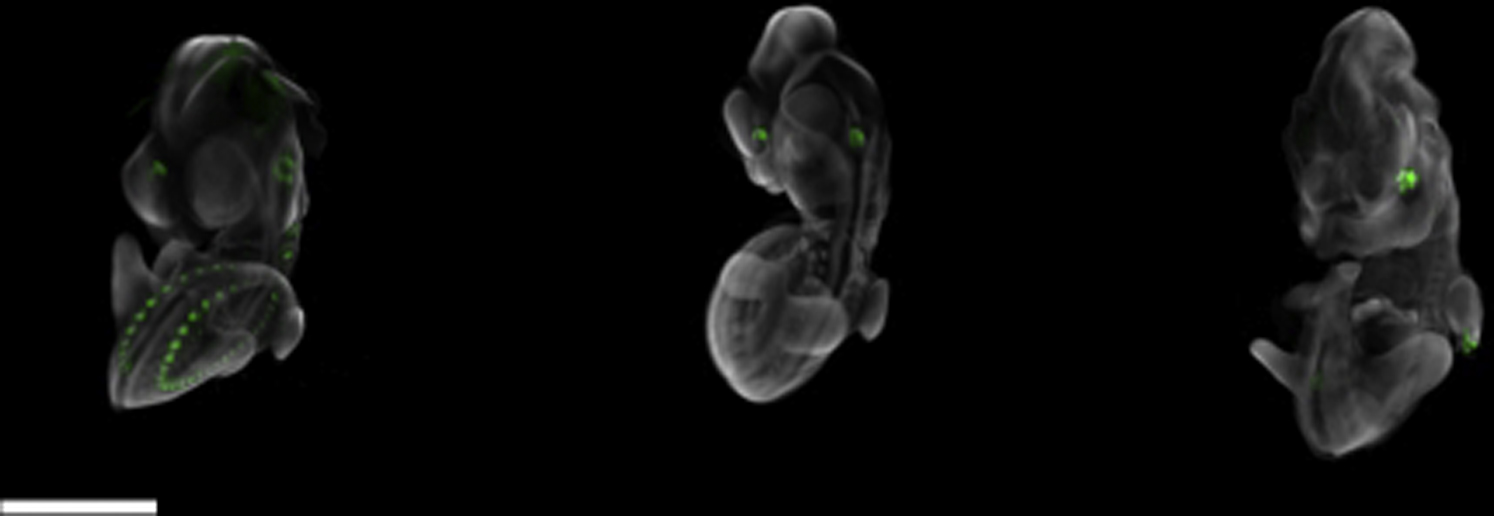

Supplement: Movie S1. OPT of LacZ-Stained E11.5 Cdkn1c-FLucLacZ Embryos, Related to Figures 1 and S1 — Absorbance (green) was measured in the developing cartilage, spine, hindbrain and liver in KImat embryos. Weaker absorbance was also detectable in liver. Very low absorbance was observed in KIpat and wt embryos. [file mmc2.jpg]
