# Supplementary material for: Visualizing Changes in Cdkn1c Expression Links Early-Life Adversity to Imprint Mis-regulation in Adults
Source: Cell Rep. 2017 Jan 31;18(5):1090–9. doi: 10.1016/j.celrep.2017.01.010 (PMC5300902; doi:10.1016/j.celrep.2017.01.010)
Supplement: Document S2. Article plus Supplemental Information [file mmc3.pdf]

Report

Cell Reports

Visualizing Changes in *Cdkn1c* Expression Links Early-Life Adversity to Imprint Mis-regulation in Adults

Graphical Abstract

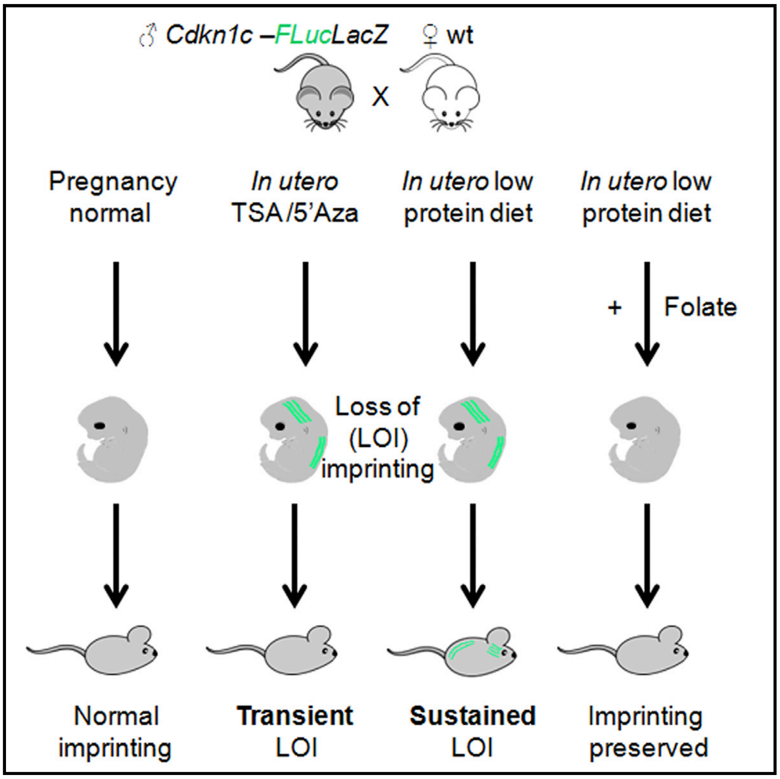

Authors

Mathew Van de Pette, Allifia Abbas, Amelie Feytout, ..., Matthias Merkenschlager, Rosalind M. John, Amanda G. Fisher

Correspondence

amanda.fisher@lms.mrc.ac.uk

In Brief

Van de Pette et al. use sensitive allele-specific reporters to longitudinally image imprinted *Cdkn1c* expression in mice and show that expression is modulated by environmental factors encountered in utero. These results establish imprinting deregulation as a mechanism linking early-life adversity to later-life outcomes and provide tools to detect imprinting changes in vivo.

Highlights

- Allele-specific expression of imprinted *Cdkn1c* imaged in vivo using bioluminescence
- Chromatin-modifying drugs applied in utero transiently de-repress *Cdkn1c* imprinting
- In utero exposure to low-protein diet permanently disrupts the *Cdkn1c* imprint
- Folate supplements during gestation protect against loss of *Cdkn1c* imprinting

Van de Pette et al., 2017, Cell Reports *18*, 1090–1099
January 31, 2017 © 2017 The Authors.
http://dx.doi.org/10.1016/j.celrep.2017.01.010

OPEN ACCESS
CellPress

Cell Reports
Report

Visualizing Changes in *Cdkn1c* Expression Links Early-Life Adversity to Imprint Mis-regulation in Adults

Mathew Van de Pette,[1] Allifia Abbas,[1] Amelie Feytout,[1] Gráinne McNamara,[1] Ludovica Bruno,[1] Wilson K. To,[1] Andrew Dimond,[1] Alessandro Sardini,[1] Zoe Webster,[1] James McGinty,[2] Eleanor J. Paul,[1] Mark A. Ungless,[1] Paul M.W. French,[2] Dominic J. Withers,[1] Anthony Uren,[1] Anne C. Ferguson-Smith,[3] Matthias Merkenschlager,[1] Rosalind M. John,[4] and Amanda G. Fisher[1,5,*]

[1]MRC London Institute of Medical Sciences, Imperial College London, Hammersmith Hospital Campus, Du Cane Road, London W12 0NN, UK
[2]Photonics Group, Department of Physics, Blackett Laboratory, Imperial College London, London SW7 2AZ, UK
[3]Department of Genetics, University of Cambridge, Downing Street, Cambridge CB2 3EH, UK
[4]Cardiff School of Biosciences, Cardiff University, Cardiff CF10 3AX, UK
[5]Lead Contact
*Correspondence: amanda.fisher@lms.mrc.ac.uk
http://dx.doi.org/10.1016/j.celrep.2017.01.010

SUMMARY

Imprinted genes are regulated according to parental origin and can influence embryonic growth and metabolism and confer disease susceptibility. Here, we designed sensitive allele-specific reporters to non-invasively monitor imprinted *Cdkn1c* expression in mice and showed that expression was modulated by environmental factors encountered in utero. Acute exposure to chromatin-modifying drugs resulted in de-repression of paternally inherited (silent) *Cdkn1c* alleles in embryos that was temporary and resolved after birth. In contrast, deprivation of maternal dietary protein in utero provoked permanent de-repression of imprinted *Cdkn1c* expression that was sustained into adulthood and occurred through a folate-dependent mechanism of DNA methylation loss. Given the function of imprinted genes in regulating behavior and metabolic processes in adults, these results establish imprinting deregulation as a credible mechanism linking early-life adversity to later-life outcomes. Furthermore, *Cdkn1c-luciferase* mice offer non-invasive tools to identify factors that disrupt epigenetic processes and strategies to limit their long-term impact.

INTRODUCTION

Epigenetics is the study of heritable changes in gene expression that arise from non-genetic influences. Genomic imprinting is an epigenetic process found in eutherian and metatherian mammals that results in parent-of-origin-specific allelic expression (John and Surani, 2000). A relatively small subset of genes within the mammalian genome (0.4%) is imprinted (Surani et al., 1984; McGrath and Solter, 1984), and these show mono-allelic expression either universally or in specific tissues that favors the maternal (e.g., *Cdkn1c* and *Ube3a*) or the paternal allele (e.g., *Dlk1* and *Nnat*; Monk et al., 2006). Imprinted expression is initially determined by differential DNA methylation that is established in the germline (Surani, 1998). Although the rationale for genomic imprinting remains uncertain, the critical role of imprinted genes in embryonic growth, placental development, and neurogenesis (Cleaton et al., 2014) suggests that imprinting may serve to balance the selective pressures between parental genomes and control in utero offspring demand (Wolf and Hager, 2006; Day and Bonduriansky, 2004; Haig, 2004). Imprinted genes encode proteins that have a wide range of roles in nutrient transport, signaling, cell-cycle control, protein synthesis and degradation, and ion channel function. Their impact extends into postnatal life with key roles in the regulation of both metabolic and neuronal processes. Alterations at imprinted gene loci in humans are associated with rare disorders, such as Beckwith-Wiedemann syndrome (Lam et al., 1999), and also more common pathological conditions, including mental disability, impaired neuro-behavioral function, diabetes, obesity, muscle hypertrophy, and also with cancer (Radford et al., 2011).

Despite their importance, imprinted genes are particularly challenging to study. This in part reflects experimental difficulties that are common to mono-allelic genes, in that gene deletion experiments show all or nothing effects, whereas alterations in gene dosage can result in complex phenotypes in which isolating genetic and epigenetic traits is problematic (Cleaton et al., 2014; John, 2010). In addition, imprinted genes are often clustered within genomic domains in which regulation is achieved through multiple levels of epigenetic control, including DNA methylation, non-coding RNAs, and modified histones (Bartolomei and Ferguson-Smith, 2011). Finally, studies to assess the impact of chromatin-modifying drugs or environmental stress on imprinted gene expression require the maternal and paternal alleles to be discriminated on the basis of heterozygous SNPs or have used LacZ-based targeting of endogenous alleles (John, 2010). Whereas such approaches provide valuable generic tools to examine imprint dynamics through development

This is an open access article under the CC BY license (http://creativecommons.org/licenses/by/4.0/).

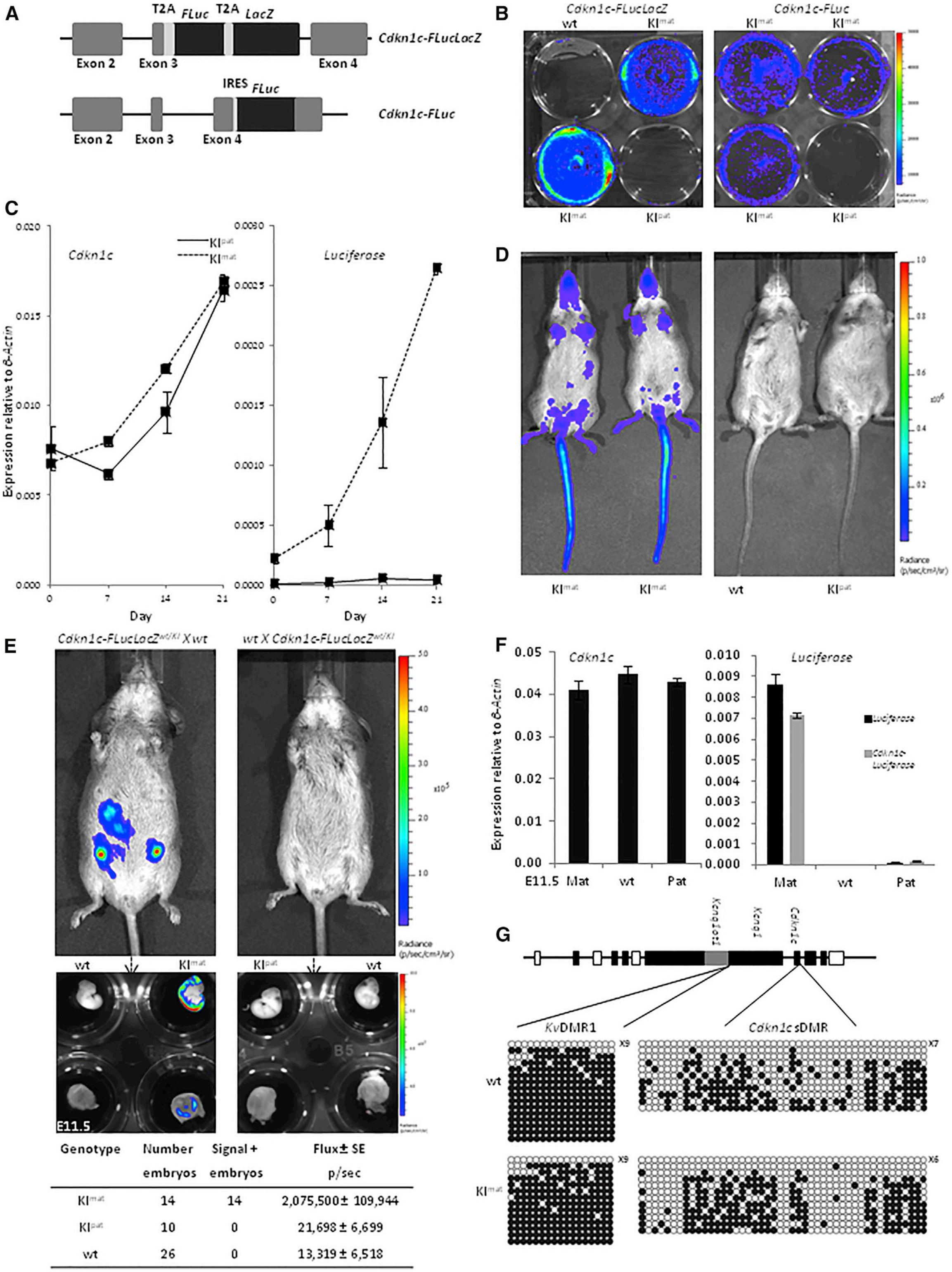

Genotype	Number embryos	Signal + embryos	Flux± SE p/sec
KI^mat	14	14	2,075,500± 109,944
KI^pat	10	0	21,698 ± 6,699
wt	26	0	13,319 ± 6,518

Figure 1. Visualizing *Cdkn1c* Gene Expression In Vivo Using Bioluminescence

(A) Scheme of alternative knockin (KI) strategies used to generate *Cdkn1c-FLucLacZ* and *Cdkn1c-FLuc* embryonic stem cells (ESCs) and reporter mouse lines, in which sequences coding for the T2A peptide, the open reading frame of FLuc, a second T2A peptide, and the open reading frame of LacZ were inserted between the last amino acid and the translation termination codon in exon 3 (…KRLREGRG…; *Cdkn1c-FLucLacZ*) or IRES elements and the open reading frame of FLuc was inserted into a unique HindIII in the 3′ UTR (*Cdkn1c-FLuc*).

(legend continued on next page)

and in response to stress, the invasive nature of the allelic readout means that it is not feasible to longitudinally monitor imprinted gene expression in different individuals throughout their life course. Models using fluorescence-based reporters have provided non-invasive readouts at whole-body and single-cell resolution (Jones et al., 2011; Swanzey and Stadtfeld, 2016), but tissue depth and sensitivity constraints may limit their general applicability. To provide new tools for investigating the consequences of early-life adversity, we generated a series of knockin embryonic stem cells (ESCs) and mouse lines in which *firefly luciferase* reports endogenous imprinted gene expression, and non-invasive bioluminescent imaging provides a means of monitoring expression longitudinally in vivo.

Cdkn1c is a maternally expressed imprinted gene that lies within the imprinting cluster 2 (IC2) on mouse chromosome 7 and is imprinted in both mice and humans (Hatada and Mukai, 1995; Hatada et al., 1996). The gene encodes a cyclin-dependent kinase inhibitor that is transiently expressed during embryogenesis in cells exiting proliferation (Lee et al., 1995; Matsuoka et al., 1995) and is particularly abundant within neural and skeleto-muscular tissue around mid-gestation (Westbury et al., 2001). *Cdkn1c* has an important role in regulating fetal growth and placental development (Andrews et al., 2007; Takahashi et al., 2000; Tunster et al., 2011) as well as lineage-specific roles, including in brown adipose tissue (Van De Pette et al., 2016), skeletal muscle (Osborn et al., 2011), and in adult quiescent stem cells (Zacharek et al., 2011; Matsumoto et al., 2011; Joseph et al., 2009). *Cdkn1c* lies within a complex imprinted domain regulated by an imprinting center that acquires DNA methylation in the maternal germline (gametic DMR; *KvDMR1*; John and Lefebvre, 2011; Hatada and Mukai, 1995; Hatada et al., 1996; Mohammad et al., 2012). This differentially methylated region spans the promoter of the paternally expressed long non-coding RNA *Kcnq1ot1* required for continuous domain-wide imprinting. The *Cdkn1c* promoter and gene body are also directly DNA methylated on the paternal allele post-fertilization, after allelic silencing has been established (somatic DMR; *Cdkn1c*-sDMR [somatic differentially methylated region]; Bhogal et al., 2004). Given the profound effect of modest dosage alteration of this gene on post-natal metabolic and behavioral processes (Andrews et al., 2007; Van De Pette et al., 2016; McNamara et al., 2016), it provides an ideal candidate to study using sensitive allele-specific reporters.

RESULTS

Generating Luciferase-Based Allelic Reporters for Mouse *Cdkn1c*

Mouse ESC lines were generated in which *firefly luciferase* (*FLuc*) alone, or in combination with *β-galactosidase* (*FLucLacZ*), was knocked into the endogenous *Cdkn1c* locus (Figures S1A and 1A, respectively). In some of the resulting targeted clones, low-level bioluminescence was detected after adding the luciferase substrate D-luciferin, consistent with insertion of *luciferase* into the maternal allele in selected clones (Figure 1B, blue). Upon differentiation, we observed increased expression of *Cdkn1c* (Figures 1C, left, and S1B, left), as anticipated from previous studies (Wood et al., 2010). In clones with a presumed maternal insertion, increased *Cdkn1c* expression was coupled to a corresponding increase in *luciferase* expression (Figures 1C and S1B). In clones with a presumed paternal insertion (KI^{pat}), increased levels of *Cdkn1c* expression were not accompanied by *luciferase* expression (Figures 1C and S1B), consistent with maintenance of the silent imprint.

Mice were generated from targeted ESCs to test whether bioluminescence was observed in offspring (Figures 1D and S1C) and to verify that this activity was transmitted in the appropriate parent-of-origin manner. Maternal transmission of the *FLucLacZ* transgene resulted in bioluminescent signal in the skin and internal organs of transgenic offspring (blue signal; KI^{mat}) at 4 weeks of age, with no signal evident in offspring after paternal inheritance (KI^{pat}) or in non-transgenic (wild-type [WT]) controls (Figure 1D). Strikingly, pregnant females carrying embryonic day 11.5 (E11.5) *Cdkn1c-FLucLacZ* KI^{mat} embryos (14/14), but not KI^{pat} embryos (0/10), showed a strong bioluminescent signal in the abdominal region (Figure 1E, upper). On dissection, transgenic embryos and placenta carrying the maternal targeted allele appropriately expressed luciferase, whereas those carrying the paternal targeted allele show no bioluminescence (Figure 1E, lower). Similar results were obtained with *Cdkn1c-FLuc* mice (Figure S1C).

(B) Low-level bioluminescence (blue-green) in *Cdkn1c-FLucLacZ* and *Cdkn1c-FLuc* ESCs was detected in clones with a presumed maternal insertion (KI^{mat}), but not in clones with a paternal insertion (KI^{pat}) or in wild-type ESCs (wt) (scale bar represents levels of bioluminescence).
(C) Total *Cdkn1c* expression (left), determined by RT-PCR, was increased in ESC clones with either a KI^{mat} (dashed line) or a KI^{pat} (solid line) insertion over 21 days of embryoid body differentiation. *Luciferase* expression (right), determined by RT-PCR, was detected uniquely in KI^{mat} clones. Samples were normalized to β-actin and expressed as the mean ± SE.
(D) Bioluminescent imaging of representative P28 female *Cdkn1c-FLucLacZ* mice. Luciferase activity was observed in *Cdkn1c-FLucLacZ* KI^{mat}, with very low/negligible signals detectable upon paternal inheritance (KI^{pat}) or in wild-type mice (wt). Strongest signal was evident in the skin, with low level signal detected in the internal organs.
(E) Bioluminescence detected in pregnancies with maternal inheritance of *Cdkn1c-FLucLacZ* (KI^{mat}, left) in utero, but not paternal inheritance (KI^{pat}, right; less than twice background). Lower panels show bioluminescence imaging of dissected E11.5 embryos, where luciferase activity was seen in head and back of KI^{mat} embryos and placental tissue and quantified (flux). All *Cdkn1c-FLucLacZ* embryos imaged showed predicted parent-of-origin-specific bioluminescent activity.
(F) Total *Cdkn1c* gene expression in embryos (E11.5) was determined by RT-PCR, and levels were similar in samples from wild-type and where *Cdkn1c-FLucLacZ* was transmitted maternally (KI^{mat}) or paternally (KI^{pat}; left). *Luciferase* (black) and *Cdkn1c-Luciferase* (gray) transcripts were detected uniquely from KI^{mat}. Samples were normalized to β-actin and expressed as the mean ± SE.
(G) Scheme of the mouse IC2 imprinting domain, showing the two DMRs that regulate *Cdkn1c* imprinted expression (*Kv*DMR1 and *Cdkn1c* sDMR) and the position of bi-allelic (white), maternally expressed (dark gray), and paternally expressed (light gray) genes. Bisulfite analysis showing DNA methylation at *Kv*DMR1 and *Cdkn1c* sDMR is similar in KI^{mat} and wt embryos at E11.5 (closed circles, methylated; open circles, un-methylated; where number indicates fully un-methylated strands).

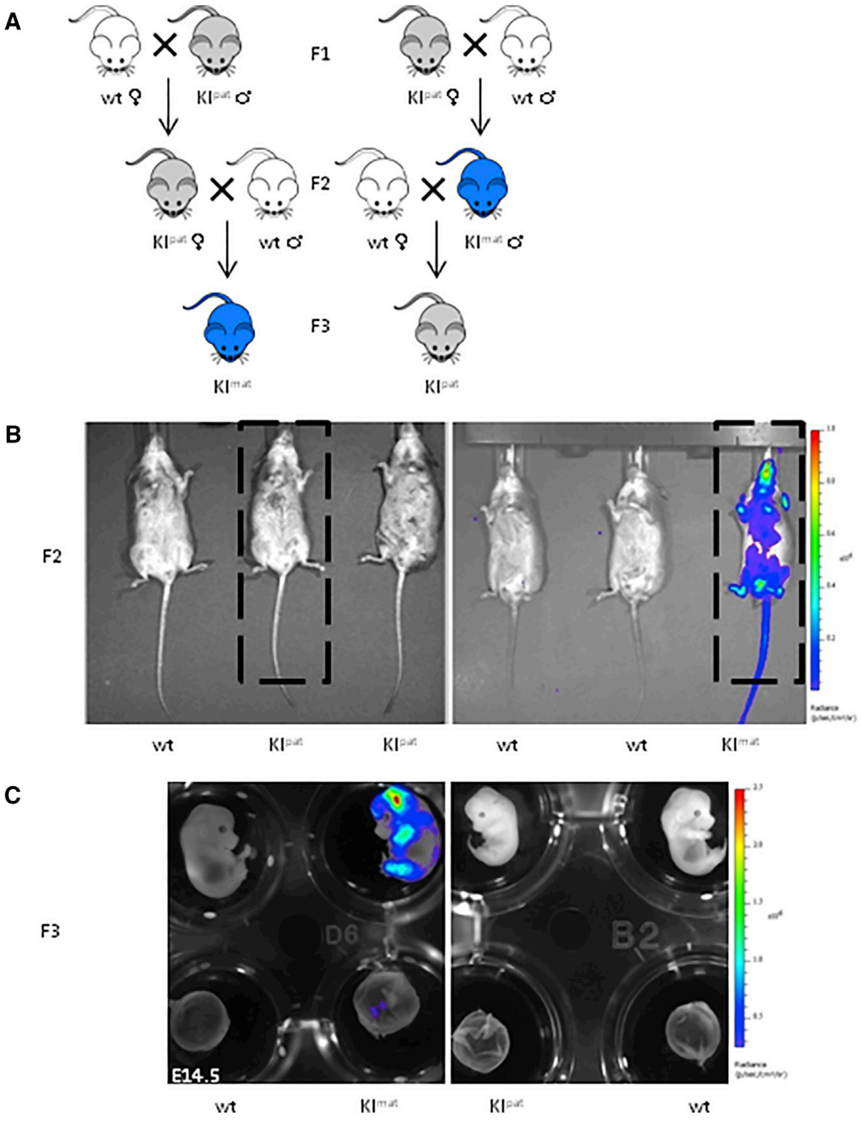

Figure 2. Correct Imprint Resetting of *Cdkn1c-FLucLacZ* across Generations

(A) Diagram showing predicted expression and inheritance of a maternally expressed imprinted gene (such as *Cdkn1c*) or transgene (*Cdkn1c-FLucLacZ*) in reciprocal crosses across three generations. Wild-type mice are shown in white, expression through maternal inheritance is shown in blue, and inheritance of a silent imprint (*Cdkn1c-FLucLacZ*; KI^{pat}) is indicated in gray.

(B) Experimental evidence of imprint resetting; bioluminescent imaging of adult F2 mice, *Cdkn1c-FLucLacZ* KI^{pat} females (left box), and KI^{mat} males (right box), showing predicted parent-of-origin-specific luciferase activity (blue). Highlighted animals were then used to generate F3 (as outlined in A).

(C) Bioluminescent image of E14.5 embryos, generated from the indicated transgenic parents; signal was detected upon maternal inheritance of *luciferase* in embryos (upper panel) and in placental tissue (lower panel; left), which had been silent in the previous generation. Conversely, paternal inheritance of the previously active luciferase was sufficient to silence the previously active allele (right).

Staining of E11.5 *Cdkn1c-FLucLacZ* KI^{mat} embryos for LacZ (Figure S1D) confirmed spatially appropriate expression in the hindbrain, spine, and developing cartilage, consistent with the published distribution of *Cdkn1c* (Westbury et al., 2001). This was further verified by 3D imaging using optical projection tomography (OPT) of cleared embryos (Figure S1D, lower; Movie S1), combined with photoacoustic tomography (Figure S1E). Importantly, no staining was detected in KI^{pat} embryos by this sensitive approach, confirming global repression of the paternal allele. Consistent with this, *luciferase* mRNA was only detectable after maternal inheritance (Figure 1F) alongside wild-type levels of the *Cdkn1c* transcript. Amplification with a primer set that spanned *Cdkn1c* exon 3 and *luciferase* exon 1 confirmed linked expression of *luciferase* and endogenous *Cdkn1c* transcripts (Figure 1F). Bisulfite analysis of the two differentially methylated regions associated with *Cdkn1c* imprinting (Bhogal et al., 2004; Mancini-Dinardo et al., 2006) showed normal DNA methylation patterns in heads of *Cdkn1c-FLucLacZ* KI^{mat} embryos (Figure 1G). Collectively, these data indicate that *luciferase* accurately reports *Cdkn1c* expression without impairing the methylation or regulation of the endogenous locus.

Imprinted *Cdkn1c-FlucLacZ* Expression Is Appropriately Reset through the Germline

Epigenetic marks that establish and maintain imprinting are normally erased and reset in the germline so that allelic expression is correctly maintained in subsequent generations (Bartolomei and Ferguson-Smith, 2011). To check whether erasure and resetting of imprints occurred normally in the *luciferase*-targeted mice, we tracked bioluminescence (blue) among reciprocal genetic crosses of *Cdkn1c-FLucLacZ* (gray) and wild-type mice (white) across generations (Figure 2A; F1, F2, and F3). Tracing bioluminescence activity across three generations revealed epigenetic inheritance as predicted (Figures 2B and 2C), in which allelic silencing of *Cdkn1c-FLucLacZ* was reversed through maternal transmission and re-established through paternal transmission. The ability to image *Cdkn1c* expression longitudinally in vivo through successive generations suggested that these reporter mice might be useful and robust models to screen for factors and environmental stresses that could interfere with imprinting. Importantly, as female mice inheriting *Cdkn1c-FLucLacZ* paternally (KI^{pat}; left box, Figure 2B) were devoid of luciferase signal, these animals offered an optimal setting (minimal background) to detect bioluminescence signals in utero from KI^{mat} embryos and placental tissue (Figures 1E, left, and 2C).

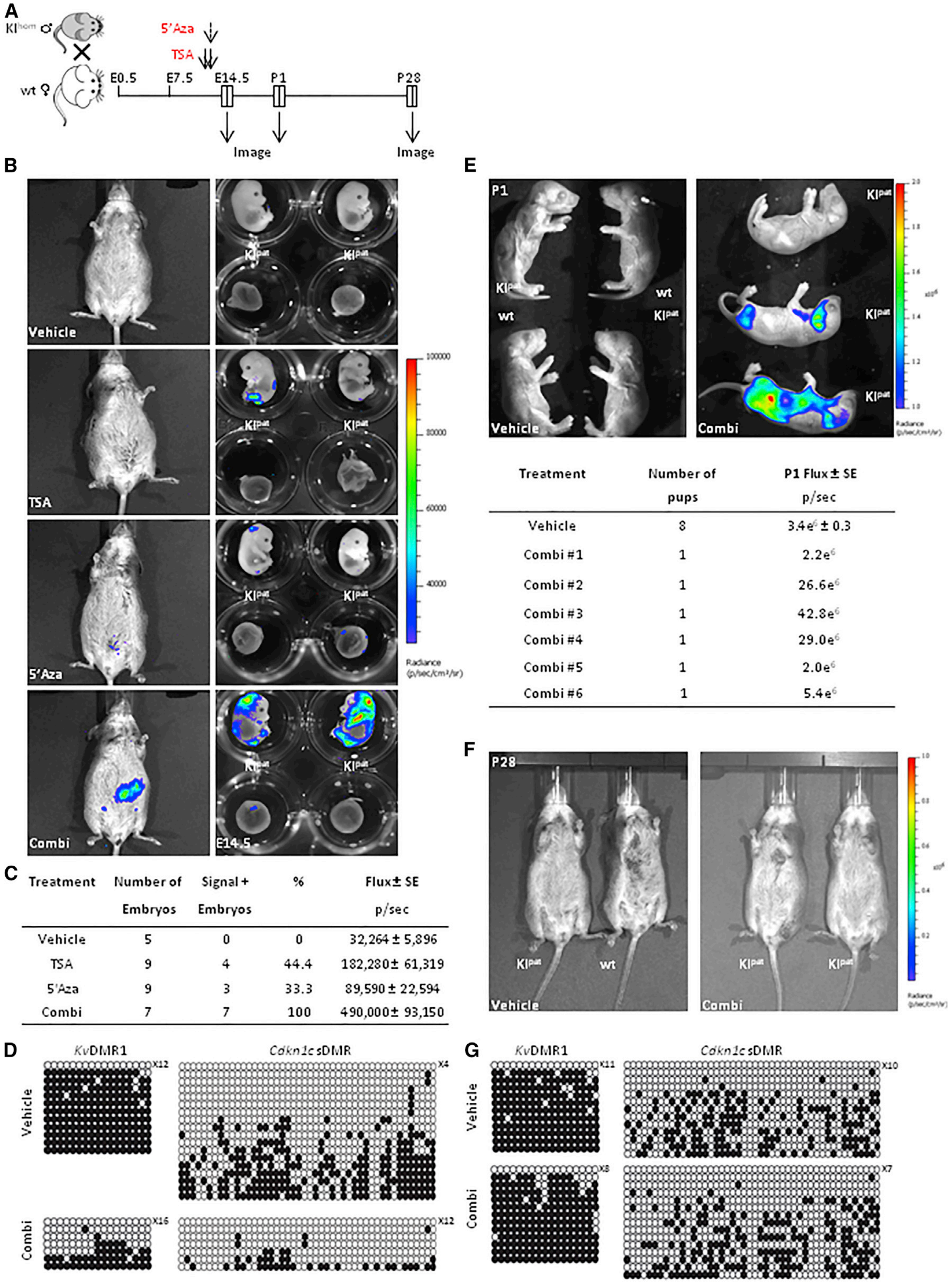

C

Treatment	Number of Embryos	Signal + Embryos	%	Flux± SE p/sec
Vehicle	5	0	0	32,264 ± 5,896
TSA	9	4	44.4	182,280± 61,319
5'Aza	9	3	33.3	89,590 ± 22,594
Combi	7	7	100	490,000± 93,150

E

Treatment	Number of pups	P1 Flux ± SE p/sec
Vehicle	8	$3.4e^6 \pm 0.3$
Combi #1	1	$2.2e^6$
Combi #2	1	$26.6e^6$
Combi #3	1	$42.8e^6$
Combi #4	1	$29.0e^6$
Combi #5	1	$2.0e^6$
Combi #6	1	$5.4e^6$

Figure 3. Silencing of Paternal *Cdkn1c-FLucLacZ* Is Transiently Released by In Utero Exposure to Epigenetic Drugs

(A) Embryos carrying silent (paternally inherited) *Cdkn1c-FLucLacZ* were generated by mating wild-type (wt) females with homozygous *Cdkn1c-FLucLacZ* males. Pregnant females were treated with trichostatin A (TSA) or 5′ azacytidine (5′Aza) alone or together at the times indicated. Offspring were imaged at E14.5, at birth (P1), and at 4 weeks of age (P28).

(legend continued on next page)

Chromatin-Modifying Drugs Transiently Disrupt Paternal Silencing of *Cdkn1c* In Utero

5′azacytidine (5′Aza) disrupts DNA methylation in cells by inhibiting DNMT1 activity, thereby preventing the incorporation of 5-methylcytosine into hemi-methylated DNA strands at S phase. In dividing cells in culture, 5′Aza treatment has been shown to reduce DNA methylation at the *Cdkn1c* promoter (Flotho et al., 2009). Trichostatin A (TSA) inhibits histone deacetylase activity and has been shown to deplete repressive histone marks at the *Cdkn1c* promoter (Yang et al., 2009). We reasoned that drugs that alter chromatin, such as 5′Aza and TSA, might be effective at disrupting *Cdkn1c* expression when epigenetic marks are consolidated (Bhogal et al., 2004; Umlauf et al., 2004). To examine this possibility, wild-type female mice were crossed with homozygous *Cdkn1c-FLucLacZ* (KI/KI) males to produce heterozygous offspring in which the *Cdkn1c-FLucLacZ* imprint was repressed. The pregnant females were then treated with drugs at E12.5–E13.5, and bioluminescence was evaluated at E14.5, at birth (P1), and at 4 weeks of age (postnatal day 28 [P28]; Figure 3A). Bioluminescence was detected in utero at E14.5 with strongest signal seen following combined drug treatment (Combi) (Figure 3B). Control vehicle-treated KI^{pat} embryos were consistently negative throughout these studies. For 5′Aza-(3/9) and TSA-treated (4/9) pregnancies, bioluminescence was not detected in all the transgenic embryos, whereas all the transgenic embryos (7/7) displayed increased luciferase activity upon combination treatment (Figure 3C). These animals showed a corresponding decrease in DNA methylation across the *Cdkn1c* somatic DMR at E14.5 as compared with controls (Figure 3D). We noticed that the levels of bioluminescence were generally lower than in age-matched *Cdkn1c-FLucLacZ* KI^{mat} embryos, consistent with partial de-repression of the paternal allele. Furthermore, de-repression appeared transient and was variable among combination drug-treated animals, as shown in pups imaged at birth (P1; Figure 3E). Four weeks after birth (P28), bioluminescence signal was no longer evident in drug-treated KI^{pat} animals (Figure 3F), and DNA methylation in the brain was similar in vehicle- and Combi-treated *Cdkn1c-FLucLacZ* KI^{pat} mice (Figure 3G). Taken together, these data show that conventional chromatin-modifying drugs alone or in combination are capable of relieving imprinted *Cdkn1c-FLucLacZ* repression in developing embryos.

Dietary Protein Restriction In Utero Provokes De-repression of Paternal *Cdkn1c* into Adulthood

Cdkn1c has previously been proposed to be sensitive to in utero dietary protein restriction (Vucetic et al., 2010). In particular, mice that were fed a low-protein diet through pregnancy (as a surrogate for early-life adversity) produced offspring with elevated levels of *Cdkn1c* in the midbrain associated with DNA hypomethylation at the promoter. To examine whether exposure to low-protein diet in utero provokes de-repression of the silent paternal *Cdkn1c-FLucLacZ*, we crossed wild-type female mice with heterozygous *Cdkn1c-FLucLacZ* (WT/KI) males (Figure 4A, schematic). Pregnant mice were fed calorie-balanced, low-protein diet (LP) from the detection of vaginal plugs until birth. All newborn offspring were maintained thereafter on a normal (unrestricted) diet. This window of exposure ensures that the influence of LP diet is restricted to a specific period of development. Although bioluminescence signal was not detected at E11.5 (Figure S2), by E14.5, all *Cdkn1c-FLucLacZ* KI^{pat} embryos expressed luciferase following maternal exposure to low-protein diet (exemplified in Figure 4A, middle right). Signal was most pronounced in the head, and luciferase re-expression among KI^{pat} embryos was prominent in the midbrain region (Figure S3). De-repression was sustained in mice imaged subsequently at 4 weeks of age (Figure 4A, lower right) and throughout adulthood, despite no longer being exposed to a restricted diet. These data establish that in utero exposure to a low-protein diet results in permanent de-repression of the normally silent paternal allele.

To further explore the mechanism underlying *Cdkn1c* re-expression, we compared DNA methylation in the brain at E11.5, E14.5, and in adults at 4 weeks of age (Figure 4B). Appropriate DNA methylation at the somatic DMR was evident at E11.5, when no luciferase activity was detected (Figure S2), but this was progressively eroded in animals exposed to LP diet during gestation (Figure 4B). These data show that, under these conditions, the somatic DMR is established correctly, but not maintained, suggesting that dietary protein may be required to sustain DNA methylation at the paternal allele. In contrast, DNA methylation at *KvDMR1* was unaffected by LP diet (Figure 4B), consistent with previous reports (Ivanova et al., 2012).

Rescue of Dietary-Induced Loss of Paternal *Cdkn1c* Silencing by Folate Supplementation

As dietary protein is known to be an important source of methyl donors required for DNA methylation, we hypothesized that a paucity of methyl donors might contribute to the failure to sustain repression of $Cdkn1c^{pat}$ alleles in vivo. To test this, we repeated the dietary experiments using the low-protein diet with increased folate supplementation as a source of methyl donors. This had a dramatic effect, reducing paternal *Cdkn1c-FLucLacZ* bioluminescence to background levels in embryos (Figure 4C, left) and in resulting adults (Figure 4C, right). We also found that, following folate supplementation, methylation of *Cdkn1c* somatic DMR

(B) Low-level bioluminescence was occasionally detected in 5′Aza- and TSA-alone treated pregnancies, whereas stronger and consistent signal (blue) was detected in combination-treated (Combi) embryos in utero (left) or individually dissected embryos (right) in the head and back.

(C) *Cdkn1c-FLucLacZ*-derived bioluminescent activity was consistently elevated in E14.5 embryos exposed to combined drug treatment.

(D) Bisulfite analysis of DNA methylation at the *Kv*DMR1 and *Cdkn1c* sDMR in the brain of E14.5 *Cdkn1c-FLucLacZ* KI^{pat} embryos shows reduced methylation in embryos exposed to combination drug treatment versus controls (closed circles, methylated; open circles, un-methylated).

(E) Variable increases in luciferase activity (blue, flux) characterize combination-drug-treated *Cdkn1c-FLucLacZ* KI^{pat} animals at P1 (right), with no signal detected in vehicle-treated controls (left).

(F) Luciferase activity was no longer detected in *Cdkn1c-FLucLacZ* KI^{pat} mice at P28 that had been exposed to combination drug treatment in utero.

(G) Bisulfite analysis of DNA methylation at the *Kv*DMR1 and *Cdkn1c* sDMR in the brain of P28 *Cdkn1c-FLucLacZ* KI^{pat} mice shows that previously ablated methylation is restored by adulthood to normal levels (closed circles, methylated; open circles, un-methylated).

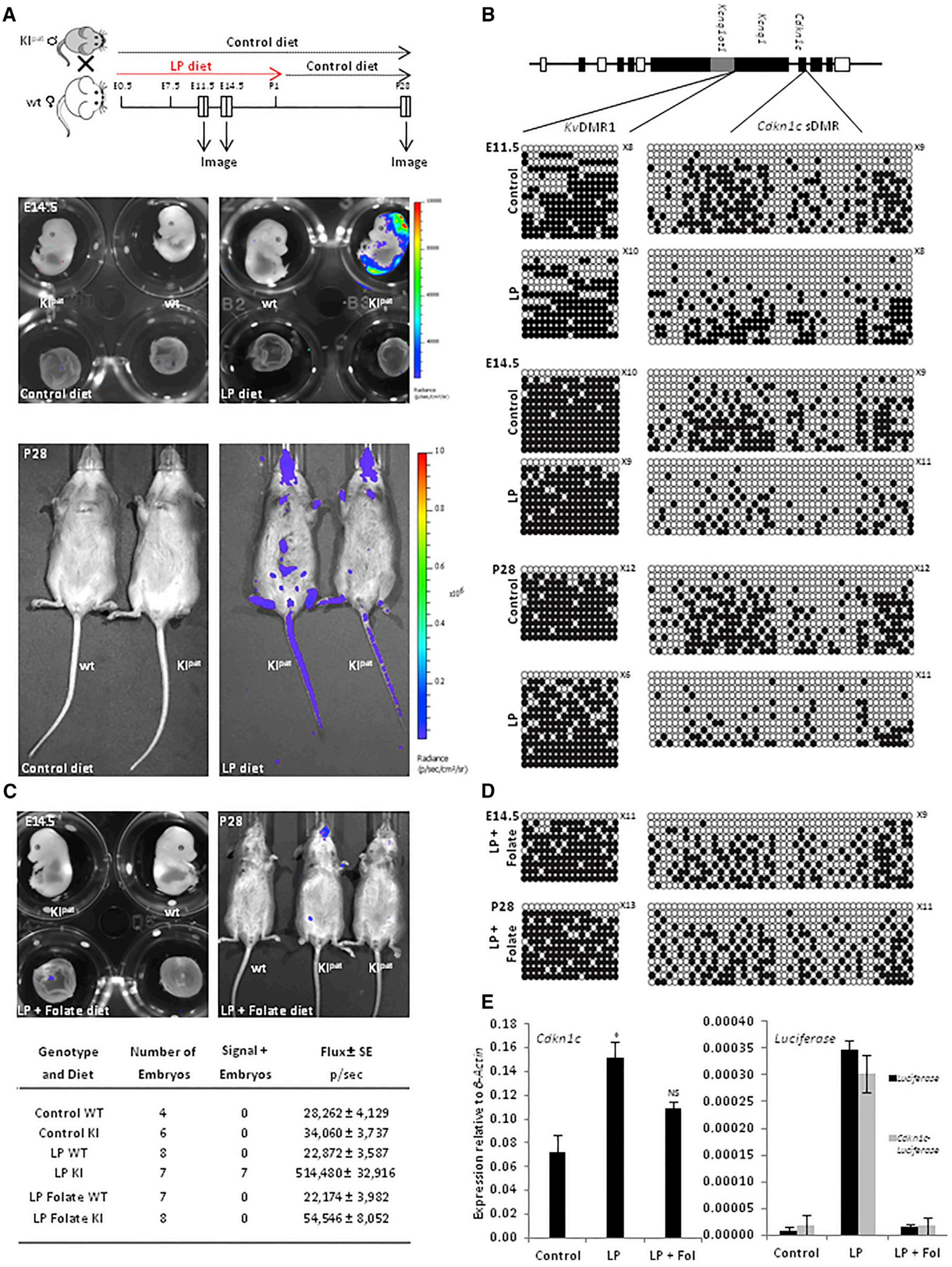

Genotype and Diet	Number of Embryos	Signal + Embryos	Flux± SE p/sec
Control WT	4	0	28,262 ± 4,129
Control KI	6	0	34,060 ± 3,737
LP WT	8	0	22,872 ± 3,587
LP KI	7	7	514,480± 32,916
LP Folate WT	7	0	22,174 ± 3,982
LP Folate KI	8	0	54,546 ± 8,052

Figure 4. Stable Silencing of Paternally Inherited *Cdkn1c-FLucLacZ* during Life Course Depends upon the Availability of Methyl Donors In Utero

(A) Offspring with a silent (paternally inherited) *Cdkn1c-FLucLacZ* were generated by mating wild-type (wt) females with heterozygous *Cdkn1c-FLucLacZ* males. Upon detection of a vaginal plug, a group of pregnant females were switched to a calorie-matched but low-protein (LP) diet for the duration of their pregnancy, with mothers and litters returning to a normal diet after birth. Pregnancies were imaged/examined at the times indicated (E11.5, E14.5, and P28). No

(legend continued on next page)

was indistinguishable from normal controls (Figures 4B and 4D). De-repression and restoration of *Cdkn1c-FlucLacZ* silencing in response to LP and LP + folate diet, respectively, was directly validated by allele-specific transcript analysis (Figure 4E). Thus, although previous studies have shown *Cdkn1c* upregulation in response to LP diet (Vucetic et al., 2010), our data now establish that dietary restriction can cause loss of imprinting.

DISCUSSION

In utero development is critically dependent on imprinted gene dose (Radford et al., 2011). This necessary control has been shown to extend into the programming of adult metabolism (Da Rocha et al., 2009; Charalambous et al., 2012). Here, we show that maternal dietary restriction has a profound impact on *Cdkn1c* expression in the embryo, provoking a partial loss of imprinting that persists through adult life, even when a normal diet is resumed. Prolonged exposure to low-protein diet during gestation erodes DNA methylation at the *Cdkn1c* somatic DMR and results in re-expression of the paternal allele. Because deregulation is rescued by elevated folate supplementation, methyl donor deprivation appears to be the most likely cause of imprint erosion. Although we do not yet know whether this reflects a specific window of vulnerability in embryonic development or simply an increased demand engendered by proliferating cells in the embryo, the observation that the gametic *Kv*DMR1 DMR resists DNA de-methylation supports previous findings that gametic differentially methylated regions (gDMRs) are relatively stable (Ivanova et al., 2012). Mechanistically, gametic and somatic DMRs both require the maintenance of DNA methylation by DNMT1 (Caspary et al., 1998; Bhogal et al., 2004). However, whereas zygotic deficiency of the de novo methylases *Dnmt3a* or *3b* has no effect on gDMR methylation, loss of *Dnmt3b* results in de-methylation of *Cdkn1c*-sDMR independent of *Kv*DMR1 status (Auclair et al., 2014). These data provide a precedent for the differential sensitivities of the gametic and somatic DMRs and implicate *Dnmt3b* as a candidate in preventing hypo-methylation at the *Cdkn1c sDMR*.

Imprinted genes are pivotal for regulating growth and metabolism, and yet the intricacies of imprinting have remained challenging to study. This reflects the intrinsic complexity of imprinting control regions (ICRs) but also a paucity of markers needed to reliably distinguish maternal from paternal alleles. Here, we describe two independent mouse lines in which luciferase-based bioluminescence reports allelic *Cdkn1c* expression, without disruption of endogenous gene output. The value of using this non-invasive approach is that it allows allelic expression to be imaged in individuals throughout life course so that epigenetic changes and their consequences can be evaluated directly. The close correspondence of luciferase expression in *Cdkn1c-FLuc* and *Cdkn1c-FLucLacZ* mouse lines suggests similar approaches might also be useful in studying allelic expression from other imprinted loci. Consistent with this idea, we have generated a series of ESC lines that report maternally expressed (*Ube3a*) or paternally expressed imprinted genes (*Dlk1*, *Nnat*, and *Igf2*) and are characterizing luciferase expression in mouse lines derived from such (Table S1). These lines, together with the *Cdkn1c-FLucLacZ* and *Cdkn1c-FLuc* lines described herein, provide novel genetic tools to interrogate the epigenetic mechanisms that establish, maintain, and reprogram imprinted gene expression in the female and the male germlines.

The observation that the *Cdkn1c* imprint is permanently disrupted by altered maternal diet provides a clear link between early-life adversity and the subsequent epigenetic mis-regulation in adult life. Our results suggest that a deficiency in methyl donor supply in utero is the most likely cause of imprint disruption, whereas limited exposure to well-characterized chromatin-modifying drugs in utero transiently deregulates imprint silencing. The basis of these different epigenetic outcomes is interesting and could reflect differences in the timing or length of exposure, inherent differences in cell proliferation, or the susceptibility of developing tissue to certain agents. Although future studies will be required to discriminate these possibilities, our ability to detect transient and permanent changes in imprint silencing in vivo offers an exciting new opportunity to explore the plasticity of epigenetic processes and their phenotypic outcome. More broadly, these luciferase-based imaging models will facilitate the rapid screening of epigenetic drugs and environmental stresses relevant for drug discovery programs and for understanding how epigenome deregulation in early life impacts upon longer-term health.

EXPERIMENTAL PROCEDURES

Animal Maintenance

Mice were handled and all in vivo studies were performed in accordance with the United Kingdom Animals (Scientific Procedures) Act (1986), were

mis-expression of luciferase was observed at day E11.5 (Figure S2), irrespective of diet; however, by E14.5, luciferase activity was detected selectively in embryos of mothers fed LP diet (upper right) and expression continued as these matured into adults (lower right). No signal was detected in animals fed normal (control) diet at any time.

(B) Comparative bisulfite analysis of DNA methylation at the *Cdkn1c* locus in the brain of *Cdkn1c-FLucLacZ* KI^{pat} animals (E11.5, E14.5, and P28) born to mothers fed control versus LP diet during pregnancy. *Cdkn1c* sDMR becomes hypo-methylated in LP conditions in utero, and methylation is not restored subsequently (closed circles, methylated; open circles, un-methylated). Methylation at the *Kv*DMR1 is unaltered.

(C) Pregnant females as in (A) were fed LP diet supplemented with increased folate. Bioluminescent imaging of embryos (E14.5) from mothers fed LP + folate showed reduced mis-expression of *Cdkn1c-FLucLacZ* KI^{pat} as compared with those fed LP alone (A), with luciferase activity remaining low or negligible as they matured into adults (P28; upper right; image scales same as A).

(D) Bisulfite analysis showing DNA methylation at *Kv*DMR1 and *Cdkn1c* sDMR in E14.5 (upper) and P28 (lower) animals born to mothers fed LP + folate diet. Progressive hypo-methylation of the *Cdkn1c* sDMR was buffered against by the increased dietary folate.

(E) Total *Cdkn1c* gene expression in E14.5 brain was determined by RT-PCR, and levels were elevated in samples from LP-exposed litters ($p < 0.033$), compared to control and LP + folate litters ($p < 0.38$). *Luciferase* (black) and *Cdkn1c-Luciferase* (gray) transcripts were detected using RT-PCR uniquely in LP brain samples, demonstrating loss of imprinting. Samples were normalized to β-actin and expressed as the mean ± SE.

approved by the Imperial College AWERB committee, and performed under a UK Home Office project license.

Epidrug Injections

5′Aza (Sigma-Aldrich) and TSA (Sigma-Aldrich) were dissolved as 0.75 μg/μL and 0.3 μg/μL stocks in PBS and 30% ethanol, respectively. Wild-type 129S2/SvHsd dams were set up with *Cdkn1c-FLucLacZ* males, and upon vaginal plug discovery, matings were separated. For 5′Aza administration, pregnant dams were injected with 5 μg/g body weight at E12.5 intraperitoneally (i.p.). For TSA administration, pregnant dams were injected i.p. with 1 μg/g body weight at E12.5 and E13.5. Vehicle injections were performed with 30% ethanol at the same time points as TSA injections. Pregnant dams and embryos were imaged at E14.5; offspring were imaged at P1 and P28.

Low Protein Study

Wild-type 129S2/SvHsd dams were set up with *Cdkn1c-FLucLacZ* males, and upon vaginal plug discovery, matings were separated. Females were fed either a low-protein chow (5769; TestDiet), a calorie-matched control chow (5755; TestDiet), or a low-protein chow with elevated folate supplement (5769 with 20 PPM Folate; TestDiet) until E11.5 or E14.5 for embryonic studies or birth for adult studies. Pregnant dams and embryos were imaged at E14.5; offspring were imaged at P28.

Bioluminescent Imaging

D-Luciferin (PerkinElmer) was dissolved in H_20 at 30 mg/mL. For in vitro studies, cells were grown to 90% confluence, 150 μg/mL was added to the medium, and plates were imaged after 2 min. For in vivo studies, mice were weighed and injected i.p. with 0.15 mg/g body weight before being anesthetized with isoflurane. Mice were imaged 10 min post-injection in an IVIS Spectrum (PerkinElmer) under anesthesia. Images of cell plates, adult mice, and pregnant dams were taken at field of view (FOV) C, with binning 4 and 180 s exposure. For imaging of embryos, pregnant females were injected with D-Luciferin at least 10 min prior to imaging. Embryos were dissected into 24-well dishes containing PBS and placed in the IVIS Spectrum. Images of embryos were taken at FOV A, with binning 1, focus 1 cm, and 180 s exposure. For epidrug and low protein imaging, settings were the same, with the exception of binning 4 in embryos. No additional D-Luciferin was added, and imaging continued for up to 35 min post-injection. Analysis of images was performed on Living Image software (Caliper Life Sciences). For quantification of bioluminescent signal, regions of interest were drawn around embryos and signal flux within the region was calculated.

SUPPLEMENTAL INFORMATION

Supplemental Information includes Supplemental Experimental Procedures, three figures, one table, and one movie and can be found with this article online at http://dx.doi.org/10.1016/j.celrep.2017.01.010.

AUTHOR CONTRIBUTIONS

M.V.d.P., R.M.J., and A.G.F. conceived of and wrote the manuscript. R.M.J., A.C.F.-S., A.U., D.J.W., and M.M. were instrumental in designing the vectors used to generate and characterize the mice. A.S., J.M., and P.M.W.F. helped with the development of imaging protocols. A.F., L.B., W.K.T., A.D., G.M., A.A., E.J.P., M.A.U., and Z.W. contributed to the experiments described.

ACKNOWLEDGMENTS

The work was funded by the MRC, the European Research Council, and institutional support to Imperial College from the Wellcome Trust, NIHR Imperial BRC (to A.A.). We thank FUJIFILM VisualSonics for their help in the development and production of photoacoustic imaging. We thank Taconic Biosciences for embryonic stem cell and animal services. We are indebted to John Savill for his advice and suggestion to develop methods allowing epigenetic change to be imaged in vivo. R.M.J.'s research is funded by MRC grant MR/M013960/1 and BBSRC grant BB/J015156. A.G.F. is funded by ERC Advanced Grant 294627.

Received: December 5, 2016
Revised: December 22, 2016
Accepted: January 7, 2017
Published: January 31, 2017

REFERENCES

Andrews, S.C., Wood, M.D., Tunster, S.J., Barton, S.C., Surani, M.A., and John, R.M. (2007). Cdkn1c (p57Kip2) is the major regulator of embryonic growth within its imprinted domain on mouse distal chromosome 7. BMC Dev. Biol. *7*, 53.

Auclair, G., Guibert, S., Bender, A., and Weber, M. (2014). Ontogeny of CpG island methylation and specificity of DNMT3 methyltransferases during embryonic development in the mouse. Genome Biol. *15*, 545.

Bartolomei, M.S., and Ferguson-Smith, A.C. (2011). Mammalian genomic imprinting. Cold Spring Harb. Perspect. Biol. *3*, a002592.

Bhogal, B., Arnaudo, A., Dymkowski, A., Best, A., and Davis, T.L. (2004). Methylation at mouse Cdkn1c is acquired during postimplantation development and functions to maintain imprinted expression. Genomics *84*, 961–970.

Caspary, T., Cleary, M.A., Baker, C.C., Guan, X.J., and Tilghman, S.M. (1998). Multiple mechanisms regulate imprinting of the mouse distal chromosome 7 gene cluster. Mol. Cell. Biol. *18*, 3466–3474.

Charalambous, M., Ferron, S.R., da Rocha, S.T., Murray, A.J., Rowland, T., Ito, M., Schuster-Gossler, K., Hernandez, A., and Ferguson-Smith, A.C. (2012). Imprinted gene dosage is critical for the transition to independent life. Cell Metab. *15*, 209–221.

Cleaton, M.A.M., Edwards, C.A., and Ferguson-Smith, A.C. (2014). Phenotypic outcomes of imprinted gene models in mice: elucidation of pre- and postnatal functions of imprinted genes. Annu. Rev. Genomics Hum. Genet. *15*, 93–126.

da Rocha, S.T., Charalambous, M., Lin, S.P., Gutteridge, I., Ito, Y., Gray, D., Dean, W., and Ferguson-Smith, A.C. (2009). Gene dosage effects of the imprinted delta-like homologue 1 (dlk1/pref1) in development: implications for the evolution of the imprinting. Plos Genetics. *5*, e1000392.

Day, T., and Bonduriansky, R. (2004). Intralocus sexual conflict can drive the evolution of genomic imprinting. Genetics *167*, 1537–1546.

Flotho, C., Claus, R., Batz, C., Schneider, M., Sandrock, I., Ihde, S., Plass, C., Niemeyer, C.M., and Lübbert, M. (2009). The DNA methyltransferase inhibitors azacitidine, decitabine and zebularine exert differential effects on cancer gene expression in acute myeloid leukemia cells. Leukemia *23*, 1019–1028.

Haig, D. (2004). Genomic imprinting and kinship: how good is the evidence? Annu. Rev. Genet. *38*, 553–585.

Hatada, I., and Mukai, T. (1995). Genomic imprinting of p57KIP2, a cyclin-dependent kinase inhibitor, in mouse. Nat. Genet. *11*, 204–206.

Hatada, I., Inazawa, J., Abe, T., Nakayama, M., Kaneko, Y., Jinno, Y., Niikawa, N., Ohashi, H., Fukushima, Y., Iida, K., et al. (1996). Genomic imprinting of human p57KIP2 and its reduced expression in Wilms' tumors. Hum. Mol. Genet. *5*, 783–788.

Ivanova, E., Chen, J.H., Segonds-Pichon, A., Ozanne, S.E., and Kelsey, G. (2012). DNA methylation at differentially methylated regions of imprinted genes is resistant to developmental programming by maternal nutrition. Epigenetics *7*, 1200–1210.

John, R.M. (2010). Engineering mouse models to investigate the function of imprinting. Brief. Funct. Genomics *9*, 294–303.

John, R.M., and Lefebvre, L. (2011). Developmental regulation of somatic imprints. Differentiation *81*, 270–280.

John, R.M., and Surani, M.A. (2000). Genomic imprinting, mammalian evolution, and the mystery of egg-laying mammals. Cell *101*, 585–588.

Jones, M.J., Bogutz, A.B., and Lefebvre, L. (2011). An extended domain of Kcnq1ot1 silencing revealed by an imprinted fluorescent reporter. Mol. Cell. Biol. *31*, 2827–2837.

Joseph, B., Andersson, E.R., Vlachos, P., Södersten, E., Liu, L., Teixeira, A.I., and Hermanson, O. (2009). p57Kip2 is a repressor of Mash1 activity and neuronal differentiation in neural stem cells. Cell Death Differ. *16*, 1256–1265.

Lam, W.W.K., Hatada, I., Ohishi, S., Mukai, T., Joyce, J.A., Cole, T.R.P., Donnai, D., Reik, W., Schofield, P.N., and Maher, E.R. (1999). Analysis of germline CDKN1C (p57KIP2) mutations in familial and sporadic Beckwith-Wiedemann syndrome (BWS) provides a novel genotype-phenotype correlation. J. Med. Genet. *36*, 518–523.

Lee, M.H., Reynisdóttir, I., and Massagué, J. (1995). Cloning of p57KIP2, a cyclin-dependent kinase inhibitor with unique domain structure and tissue distribution. Genes Dev. *9*, 639–649.

Mancini-Dinardo, D., Steele, S.J.S., Levorse, J.M., Ingram, R.S., and Tilghman, S.M. (2006). Elongation of the Kcnq1ot1 transcript is required for genomic imprinting of neighboring genes. Genes Dev. *20*, 1268–1282.

Matsumoto, A., Takeishi, S., Kanie, T., Susaki, E., Onoyama, I., Tateishi, Y., Nakayama, K., and Nakayama, K.I. (2011). p57 is required for quiescence and maintenance of adult hematopoietic stem cells. Cell Stem Cell *9*, 262–271.

Matsuoka, S., Edwards, M.C., Bai, C., Parker, S., Zhang, P., Baldini, A., Harper, J.W., and Elledge, S.J. (1995). p57KIP2, a structurally distinct member of the p21CIP1 Cdk inhibitor family, is a candidate tumor suppressor gene. Genes Dev. *9*, 650–662.

McGrath, J., and Solter, D. (1984). Completion of mouse embryogenesis requires both the maternal and paternal genomes. Cell *37*, 179–183.

McNamara, G.I., Davis, B.A., Dwyer, D.M., John, R.M., and Isles, A.R. (2016). Behavioural abnormalities in a novel mouse model for Silver Russell syndrome. Hum. Mol. Genet. Published online October 24, 2016. http://dx.doi.org/10.1093/hmg/ddw357.

Mohammad, F., Pandey, G.K., Mondal, T., Enroth, S., Redrup, L., Gyllensten, U., and Kanduri, C. (2012). Long noncoding RNA-mediated maintenance of DNA methylation and transcriptional gene silencing. Development *139*, 2792–2803.

Monk, D., Arnaud, P., Apostolidou, S., Hills, F.A., Kelsey, G., Stanier, P., Feil, R., and Moore, G.E. (2006). Limited evolutionary conservation of imprinting in the human placenta. Proc. Natl. Acad. Sci. USA *103*, 6623–6628.

Osborn, D.P.S., Li, K., Hinits, Y., and Hughes, S.M. (2011). Cdkn1c drives muscle differentiation through a positive feedback loop with Myod. Dev. Biol. *350*, 464–475.

Radford, E.J., Ferrón, S.R., and Ferguson-Smith, A.C. (2011). Genomic imprinting as an adaptative model of developmental plasticity. FEBS Lett. *585*, 2059–2066.

Surani, M.A. (1998). Imprinting and the initiation of gene silencing in the germ line. Cell *93*, 309–312.

Surani, M.A.H., Barton, S.C., and Norris, M.L. (1984). Development of reconstituted mouse eggs suggests imprinting of the genome during gametogenesis. Nature *308*, 548–550.

Swanzey, E., and Stadtfeld, M. (2016). A reporter model to visualize imprinting stability at the Dlk1 locus during mouse development and in pluripotent cells. Development *143*, 4161–4166.

Takahashi, K., Nakayama, K., and Nakayama, K. (2000). Mice lacking a CDK inhibitor, p57Kip2, exhibit skeletal abnormalities and growth retardation. J. Biochem. *127*, 73–83.

Tunster, S.J., Van de Pette, M., and John, R.M. (2011). Fetal overgrowth in the Cdkn1c mouse model of Beckwith-Wiedemann syndrome. Dis. Model. Mech. *4*, 814–821.

Umlauf, D., Goto, Y., Cao, R., Cerqueira, F., Wagschal, A., Zhang, Y., and Feil, R. (2004). Imprinting along the Kcnq1 domain on mouse chromosome 7 involves repressive histone methylation and recruitment of Polycomb group complexes. Nat. Genet. *36*, 1296–1300.

Van De Pette, M., Tunster, S.J., McNamara, G.I., Shelkovnikova, T., Millership, S., Benson, L., Peirson, S., Christian, M., Vidal-Puig, A., and John, R.M. (2016). Cdkn1c boosts the development of brown adipose tissue in a murine model of Silver Russell syndrome. PLoS Genet. *12*, e1005916.

Vucetic, Z., Totoki, K., Schoch, H., Whitaker, K.W., Hill-Smith, T., Lucki, I., and Reyes, T.M. (2010). Early life protein restriction alters dopamine circuitry. Neuroscience *168*, 359–370.

Westbury, J., Watkins, M., Ferguson-Smith, A.C., and Smith, J. (2001). Dynamic temporal and spatial regulation of the cdk inhibitor p57(kip2) during embryo morphogenesis. Mech. Dev. *109*, 83–89.

Wolf, J.B., and Hager, R. (2006). A maternal-offspring coadaptation theory for the evolution of genomic imprinting. PLoS Biol. *4*, e380.

Wood, M.D., Hiura, H., Tunster, S.J., Arima, T., Shin, J.Y., Higgins, M.J., and John, R.M. (2010). Autonomous silencing of the imprinted Cdkn1c gene in stem cells. Epigenetics *5*, 214–221.

Yang, X., Murthy Karuturi, R.K., Sun, F., Aau, M., Yu, K., Shao, R., Miller, L.D., Boon Ooi Tan, P., and Yu, Q. (2009). *CDKN1C* ($p57^{KIP2}$) is a direct target of EZH2 and suppressed by multiple epigenetic mechanisms in breast cancer cells. PLoS One *4*, e5011.

Zacharek, S.J., Fillmore, C.M., Lau, A.N., Gludish, D.W., Chou, A., Ho, J.W., Zamponi, R., Gazit, R., Bock, C., Jäger, N., et al. (2011). Lung stem cell self-renewal relies on BMI1-dependent control of expression at imprinted loci. Cell Stem Cell *9*, 272–281.

Cell Reports, Volume *18*

Supplemental Information

Visualizing Changes in *Cdkn1c* Expression Links Early-Life Adversity to Imprint Mis-regulation in Adults

Mathew Van de Pette, Allifia Abbas, Amelie Feytout, Gráinne McNamara, Ludovica Bruno, Wilson K. To, Andrew Dimond, Alessandro Sardini, Zoe Webster, James McGinty, Eleanor J. Paul, Mark A. Ungless, Paul M.W. French, Dominic J. Withers, Anthony Uren, Anne C. Ferguson-Smith, Matthias Merkenschlager, Rosalind M. John, and Amanda G. Fisher

Supplementary figures

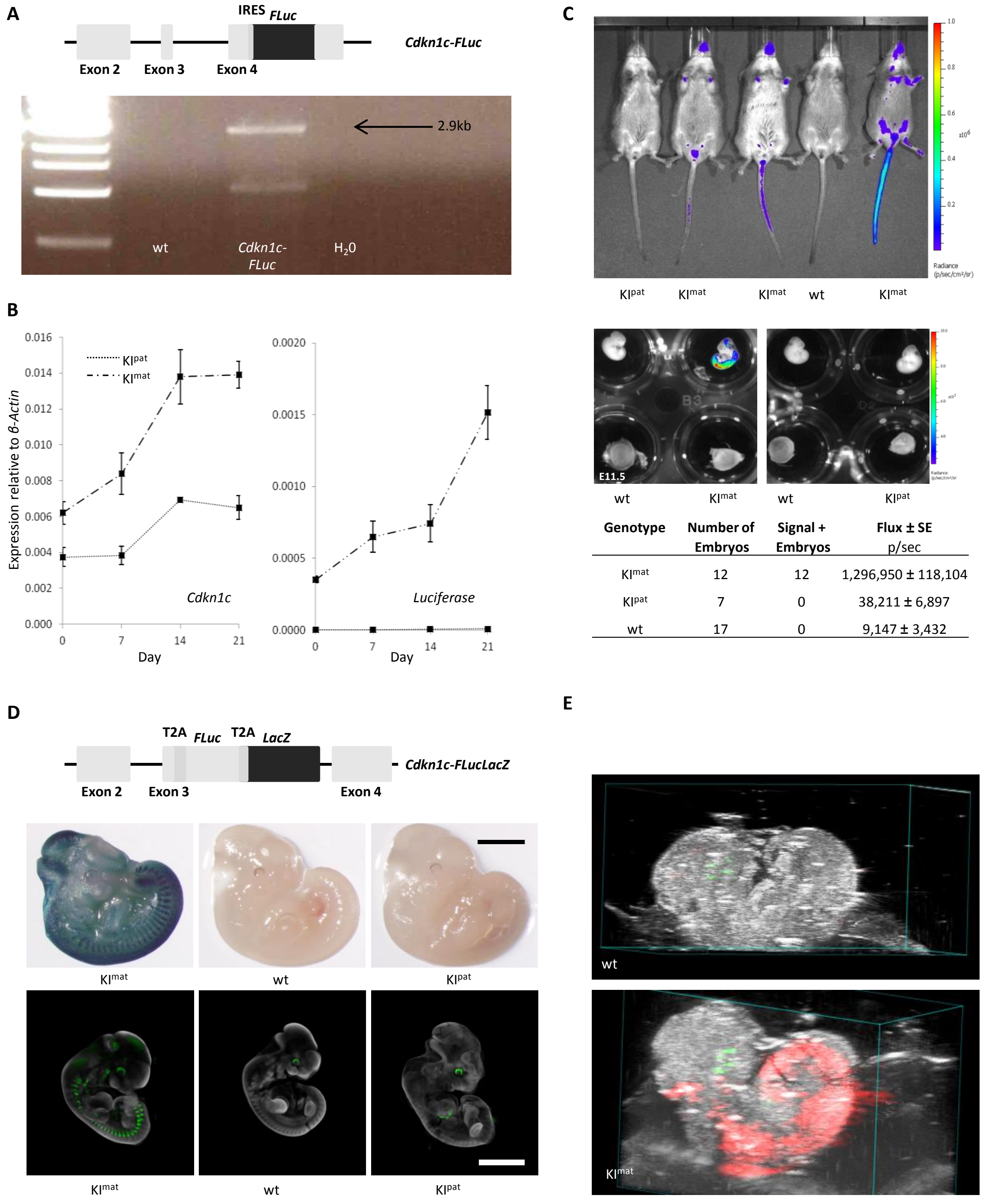

Figure S1. Characterization of *Cdkn1c-FLuc* and *Cdkn1c-FLucLacZ* lines, related to Figure 1.

(A) Construct designed to generate *Cdkn1c-FLuc* reporter ESCs, where insertion within the endogenous *Cdkn1c locus* was verified by long-range PCR and confirmed by DNA sequencing; 2.9 kb fragment corresponding to the *luciferase* transgene was detected in the targeted clone, not in wild type (wt) control cells. **(B)** Upon

differentiation to embryoid bodies total *Cdkn1c* transcript levels increased, as detected by RT-PCR, in clones that carried either a maternal (large dash) or a paternal (small dash) insertion. *Luciferase* transcripts, in contrast, were uniquely detected in ESC clones that had *Cdkn1c-FLuc* inserted into the maternal locus. **(C)** Bioluminescence imaging of representative P28 female *Cdkn1c-FLuc* mice shows signal (blue) in mice inheriting *luciferase* KI maternally (KI^{mat}), and no expression in wild type (wt) controls or animals with paternal inheritance (KI^{pat}). Bioluminescent signal (green, flux) in E11.5 embryos upon maternal inheritance but not paternal inheritance of *Cdkn1c-FLuc.* **(D)** Construct designed to generate *Cdkn1c-FLucLacZ* reporter ESCs (as in Figure 1A). LacZ staining of *Cdkn1c-FLucLacZ* embryos at E11.5 showing labeling of cartilage, spine and hind brain in KI^{mat} embryos, with no staining detected in KI^{pat} or wt embryos. Scale bar: 2 mm. Optical Projection Tomography (OPT) of LacZ stained E11.5 *Cdkn1c-FLucLacZ* embryos. Absorbance (green) was measured in the developing cartilage, spine, hindbrain and liver of KI^{mat} embryos. Very low absorbance was observed in KI^{pat} and wt embryos. Scale bar: 2 mm. Videos available of 3D reconstructions. **(E)** Photoacoustic imaging of LacZ stained E11.5 wt and KI^{mat} *Cdkn1c-FLucLacZ* embryos; signal (red) overlays ultrasound (grey) and shows detection in cartilage, spine, hind brain and liver.

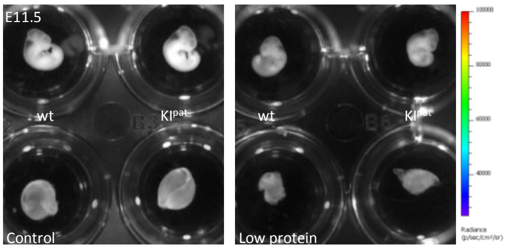

Genotype and Diet	Number of Embryos	Number+ Embryos	Flux ± SE p/sec
Control WT	6	0	14,395 ± 5,229
Control KI	5	0	26,438 ± 5947
Low protein WT	6	0	12,812 ± 7,680
Low protein KI	8	0	28,280 ± 7,908

Figure S2. Silencing of paternal *Cdkn1c-FLucLacZ* is retained at E11.5 in embryos exposed to LP diet *in utero*, related to Figure 4.

Cdkn1c-FLucLacZ KI^{pat} embryos were generated as in Figure 4A, with mothers receiving either normal (control) or low protein (LP) diet throughout pregnancy. Luciferase activity remained low in all *Cdkn1c-FLucLacZ* KI^{pat} embryos at E11.5, consistent with correct imprinting of the paternal allele at this stage.

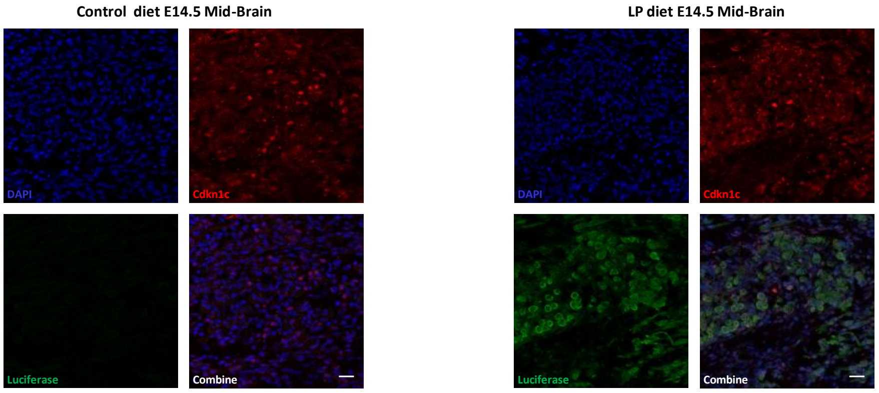

Figure S3. Low protein diet *in utero* disrupts *Cdkn1c* KI^{pat} silencing in regions of the brain, related to Figure 4.

(A) Cdkn1c (red) and luciferase (green) immunostaining in the E14.5 *Cdkn1c-FLucLacZ* KI^{pat} midbrain, where cell nuclei are highlighted by DAPI (blue). Embryos exposed to maternal low protein diet show loss of imprinting in the midbrain, with negligible signal detected in control embryos. Scale bar: 30 µm.

Table S1. Bioluminescent Reporters of Imprinted Gene Expression Models (BRIGEM) embryonic stem cell lines, related to Figure 1 and Figure S1.

Imprinted gene	Parental expression	ICR gametic methylation	Somatic DMR	ESC targeted line	No. of independent clones
Cdkn1c	Maternal	Maternal (*Kv*DMR1, *Kcnq1ot1* promoter)	*Cdkn1c* promoter (Paternal)	*Cdkn1c-T2A-FLuc-T2A-LacZ*	2
				Cdkn1c-IRES-Fluc	6
Dlk1	Paternal	Paternal (IG-DMR, intergenic)	No	*Dlk1-T2A-FLuc-T2A-LacZ*	1
Nnat	Paternal	Maternal (*Nnat* promoter)	No	*FLuc-T2A-LacZ-T2A-Nnat*	2
Igf2	Paternal	Paternal (*H19*-DMD, intergenic)	Igf2-DMR0 (Maternal, placenta only) Igf2-DMR1 (Paternal) Igf2-DMR2 (Paternal)	*Igf2-T2A-Luc2-T2A-LacZ*	2
Ube3a	Maternal (only in brain; bi-allelic in other tissues)	Maternal (*Snrpn*-CGI, *Snrpn* promoter)	No	*Ube3a-T2A-Luc2*	2

Video S1, related to Figure 1.

OPT of LacZ stained E11.5 *Cdkn1c-FLucLacZ* embryos. Absorbance (green) was measured in the developing cartilage, spine, hindbrain and liver in KI^{mat} embryos. Weaker absorbance was also detectable in liver. Very low absorbance was observed in KI^{pat} and wt embryos.

Supplemental Experimental Procedures

Generation of targeted ESCs and mice

The *Cdkn1c-IRES-luciferase* targeting sequence (*Cdkn1c-Fluc*) was generated in the pPGKneobpAlox2PGKDTA vector (Soriano's lab) by restriction fragment cloning of *IRES-Fluc* (Addgene) into the *Cdkn1c* 3'UTR (*HindIII* site) along with 2.9 kb of upstream sequence and 2.85 kb of *Cdkn1c* genomic sequence. Targeting was confirmed by long range PCR (Figure S1A). The *Cdkn1c-FLucLacZ* line was created by Taconic Biosciences and ESCs and animal founders were delivered to Imperial College. The ESC lines presented in Table S1 were also created by Taconic Biosciences.

ESC culture and embryoid body differentiation

ESCs were maintained in knockout DMEM (Gibco) supplemented with 10% foetal bovine serum (Gibco), penicillin/streptomycin, β-mercaptoethanol, L-Glutamine and LIF, on gelatinised plates, as previously described (Pereira et al., 2008). For embryoid body differentiation, cells were plated onto low adherence plates without LIF. Medium was changed every 1-2 days and samples were taken at the indicated time points.

Maintenance of mice

Mice were handled and all *in vivo* studies were performed in accordance with the United Kingdom Animals (Scientific Procedures) Act (1986), were approved by the Imperial College AWERB committee and performed under a UK Home Office project license. Mice were housed on a 12 hour light-dark cycle with a temperature range of 21 +/- 2°C in pathogen free conditions. *Cdkn1c-FLucLacZ* and *Cdkn1c-FLuc* lines were maintained on a 129S2/SvHsd background. For mating, males were set up with not more than three females and morning plug checking was performed. Upon plug discovery, females were considered E0.5.

Genotyping of animals

Genomic DNA was isolated from 4-week old ear biopsies or embryonic tails by digestion in lysis buffer (0.05 M Tris HCl pH 8, 0.025 M EDTA, 0.031% SDS, 0.02 M NaCl, 80 µg/ml Proteinase K (Sigma-Aldrich)) at 50°C with rocking. DNA was diluted 1:2 in 10 mM Tris HCl pH8 and 1 µl of diluted DNA was used in PCR analysis.

Beta-Galactosidase staining

E11.5 embryos were dissected and placed in cold LacZ fixative (2% formaldehyde, 0.2% glutaraldehyde, 0.02% Nonidet P-40, 1 mM $MgCl_2$, 0.1 mg/ml Sodium Deoxycholate in PBS) for 1 hour, kept at 4°C with rocking. Tissue was washed in PBS before being placed in LacZ stain (0.4 mg/ml X-Gal, 4 mM Potassium Ferrocyanide, 4 mM Potassium Ferricyanide, 1 mM $MgCl_2$, 0.02% Nonidet P-40 in PBS) for 4-6 hours at 4°C with rocking. Upon completion, embryos were washed twice in PBS before transfer to 70% ethanol and storage at 4°C. Photography was performed under standard light field conditions.

Optical projection tomography

LacZ stained E11.5 embryos (as above) were mounted in cylinders of 2% low melting point agarose. The mounted samples were dehydrated through graded methanol solutions and maintained in 100% methanol prior to clearing. They were subsequently immersed overnight in an optical clearing solution, BABB (1:2 Benzyl benzoate: Benzyl alcohol, Sigma Aldrich). Optical projection tomography (OPT) (Sharpe et al., 2002) was performed on a low-magnification imaging system. Briefly, the cleared samples were suspended from a rotation

stage (T-NM17A200, Zaber Technologies Inc) in a cuvette filled with BABB and imaged using a telecentric zoom lens (modules NT56-625, NT59-671 and NT59-672, Edmund Optics Ltd) with images recorded using a CCD camera operated at 2×2 pixel binning (Clara, Andor Technology Ltd). To measure the distribution of LacZ staining, transmitted light images were acquired every 1° over a full 360° sample rotation through a 716±20 nm band-pass filter (FF01-716/40-25, Laser 2000 UK Ltd). Average illumination and background images were also acquired. At each projection angle these images were combined to form an integrated absorption coefficient image, given by

$$\sum_i \alpha_i = \frac{1}{\Delta l} \ln \left| \frac{I_0 - I_b}{I - I_b} \right|$$

where $\sum_i \alpha_i$ is the sum of absorption coefficients from the voxels along a 'line-of-sight' (i.e. a projection), Δl is the linear size of a voxel (18.7 μm), I is the transmitted light image (i.e. image of the sample), I_0 is the average illumination image and I_b is the average background image. A filtered back-projection algorithm (Kak, 1988) was applied to this transformed dataset to produce a 3-D reconstruction of the absorption coefficient per voxel (displayed in green). In addition to the transmitted light data, fluorescence OPT acquisitions using a 473 nm excitation source (Cobolt Blues^{TM}, Cobolt AB) imaging at 520±17 nm (FF01-520/35-25, Laser 2000 UK Ltd) were also performed to reconstruct the whole sample volume (shown in greyscale).

Photoacoustic tomography

LacZ stained E11.5 embryos (as above, without clearing) were immersed in ultrasound gel and imaged by ultrasound and photoacoustic tomography with a Vevo-LAZR micro-ultrasound imaging system (FUJIFILM VisualSonics) using a 40 MHz centre frequency probe (LZ 550). The linear array transducer had a bandwidth of 32-55 MHz, axial resolution of 40 µm and lateral resolution of 90 µm. Photoacoustic spectra of the embryos were obtained by performing spectral scans from 680 nm to 970 nm with 5 nm step size. Three dimensional multi-spectral unmixing was performed using 680 nm, 720 nm, 750 nm, 800 nm and 850 nm laser wavelengths. For generation of stacks, a total volume of 8 mm was scanned, with z step size of 0.076 mm.

Immunofluorescence

Embryos were dissected and fixed for 4 hours in phosphate-buffered 4% paraformaldehyde. Samples were washed in PBS and transferred to 30% sucrose in PBS for cryopreservation. After cryopreservation, samples were embedded in OCT and stored at -80°C until use. Sections of 10 µm were cut with a cryostat (Leica) and applied to polysine slides (VWR). Circles were drawn around sections with a hydrophobic pen (Invitrogen) and sections were incubated in block solution (5% Donkey Serum, 0.1% Fraction V BSA, 0.1% Triton-X100 (all Sigma-Aldrich)) for 30 mins. After blocking, immunolabelling for Cdkn1c (1:200, KP39, SantaCruz) and Luciferase (1:250, L0159, Sigma-Aldrich) was carried out and detected using Alexa Fluor® conjugated secondary antibodies (Abcam). Slides were mounted with DAPI containing medium (Vector) and images were acquired using a Leica SP8 confocal microscope and LAS X software.

RNA extraction and RT-PCR analysis

RNA was extracted with RNA-Bee (Amsbio) and all RNA precipitation steps were performed with 100% ethanol. Reverse transcription was performed using Superscript III Reverse transcriptase (Invitrogen) as per the manufacturer's protocol, with minor modifications. RT-PCR was performed on a CFX96 Real-Time System (Bio-Rad) with QuantiTect SYBR Green Master Mix (Qiagen) as per the manufacturer's protocol. Samples were normalised to *β-Actin* and expressed as the mean ± standard error. Student's T-test was performed for statistical analysis of *Cdkn1*c expression in low protein study.

Bisulphite sequencing

Bisulphite modification of DNA was carried out with the EZ DNA Methylation Kit (ZymoGenetics) according to the manufacturer's recommendations. PCR primers that specifically recognize bisulphite-converted DNA were used to amplify regions spanning three imprinted DMRs. PCR products were separated on an agarose gel and bands corresponding to the predicted size were excised and cleaned up with a Gel Extraction kit (QIAquick, Qiagen). Ligation of product into pGEM-T Easy vector (Promega) was performed before transformation into DH5alpha cells. Cells were plated onto LB/Ampicillin/IPTG/X-Gal plates and grown up overnight at 37 °C.

Colonies were picked (24 per sample) and expanded in LB/Ampicillin broth overnight at 37°C. The following morning, plasmids were purified with the Wizard® SV 96 Plasmid DNA Purification System (Promega) according to the manufacturer's recommendations and sent for sequencing.

Bisulphite primers

Cdkn1c sDMR F: AGTATAATGTAGTATTTTTAGT
Cdkn1c sDMR R: AAAACTATACCCAACTCCATA

*Kv*DMR1 F: TAAGGTGAGTGGTTTAGGAT
*Kv*DMR1 OutR: AATCCCCCACACCTAAATTC
*Kv*DMR1 InR: CCACTATAAACCCACACATA

M13 R: CAGGAAACAGCTATGAC

Primers

5'Long Range *Cdkn1c-FLuc* F: CCAGGACCCAGCTGGTAGTA
5'Long Range *Cdkn1c-FLuc* R: AGGAACTGCTTCCTTCACGA

3'Long Range *Cdkn1c-Fluc* F: GCTTCTGAGGCGGAAAGAAC
3'Long Range *Cdkn1c-Fluc* R: GGGGCCTGAATTGCAACTTA

Cdkn1c-FLucLacZ KI GenoF: CTCCATGCGATCACAGTGG
Cdkn1c-FLucLacZ KI GenoR: CTTTGGATCCAGTGGACTGG

β-Actin F: CCTGTATGCCTCTGGTCGTA
β-Actin R: CCATCTCCTGCTCGAAGTCT

Luciferase F: GTTTTGGAGCACGGAAAGAC
Luciferase R: ACCTTTCGGTACTTCGTCCA

Cdkn1c F: AGAGAACTGCGCAGGAGAAC
Cdkn1c R: TCTGGCCGTTAGCCTCTAAA

Cdkn1c-FLuc F: AGAGAACTGCGCAGGAGAAC
Cdkn1c-FLuc R: GTTCCATCTTCCAGCGGATA

General experimental approaches

Where possible, investigators were blinded to the genotype of both study animals and that of tissue samples. Treatments were administered in random order, with all pharmacological and metabolic studies replicated in at least two independent cohorts.

KAK, A. C. S., M; 1988. Principles of computerized tomographic imaging. IEEE Press, New York.
PEREIRA, C. F., TERRANOVA, R., RYAN, N. K., SANTOS, J., MORRIS, K. J., CUI, W., MERKENSCHLAGER, M. & FISHER, A. G. 2008. Heterokaryon-Based Reprogramming of Human B Lymphocytes for Pluripotency Requires Oct4 but Not Sox2. *Plos Genetics*, 4.
SHARPE, J., AHLGREN, U., PERRY, P., HILL, B., ROSS, A., HECKSHER-SORENSEN, J., BALDOCK, R. & DAVIDSON, D. 2002. Optical projection tomography as a tool for 3D microscopy and gene expression studies. *Science*, 296**,** 541-545.
